# In Vivo Photopolymerization: Achieving Detailed Conducting Patterns for Bioelectronics

**DOI:** 10.1002/advs.202408628

**Published:** 2024-11-07

**Authors:** Fredrik Ek, Tobias Abrahamsson, Marios Savvakis, Stefan Bormann, Abdelrazek H. Mousa, Muhammad Anwar Shameem, Karin Hellman, Amit Singh Yadav, Lazaro Hiram Betancourt, Peter Ekström, Jennifer Y. Gerasimov, Daniel T. Simon, György Marko‐Varga, Martin Hjort, Magnus Berggren, Xenofon Strakosas, Roger Olsson

**Affiliations:** ^1^ Chemical Biology & Therapeutics Department of Experimental Medical Science Lund University Lund SE‐221 84 Sweden; ^2^ Laboratory of Organic Electronics Department of Science and Technology Linköping University Norrköping 601 74 Sweden; ^3^ Department of Chemistry and Molecular Biology University of Gothenburg Gothenburg SE‐405 30 Sweden; ^4^ Division of Oncology Department of Clinical Sciences Lund Lund University Lund SE‐221 84 Sweden; ^5^ Wallenberg Initiative Materials Science for Sustainability, Department of Science and Technology Linköping University Norrköping 60174 Sweden

**Keywords:** biocompatibility, bioelectronics, in vivo, photolithography, photopolymerization

## Abstract

Bioelectronics holds great potential as therapeutics, but introducing conductive structures within the body poses great challenges. While implanted rigid and substrate‐bound electrodes often result in inflammation and scarring in vivo, they outperform the in situ‐formed, more biocompatible electrodes by providing superior control over electrode geometry. For example, one of the most researched methodologies, the formation of conductive polymers through enzymatic catalysis in vivo, is governed by diffusion control due to the slow kinetics, with curing times that span several hours to days. Herein, the discovery of the formation of biocompatible conductive structures through photopolymerization in vivo, enabling spatial control of electrode patterns is reported. The process involves photopolymerizing novel photoactive monomers, 3Es (EDOT‐trimers) alone and in a mixture to cure the poly(3, 4‐ethylenedioxythiophene)butoxy‐1‐sulfonate (PEDOT‐S) derivative A5, resulting in conductive structures defined by photolithography masks. These reactions are adapted to in vivo conditions using green and red lights, with short curing times of 5–30 min. In contrast to the basic electrode structures formed through other in situ methods, the formation of specific and layered patterns is shown. This opens up the creation of more complex 3D layers‐on‐layer circuits in vivo.

## Graphical abstract

Formation of biocompatible conductive structures in vivo through photopolymerization, enabling structural control of electrode patterns.

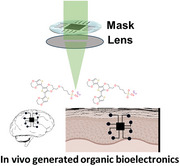

## 1 Introduction

Integrating electronics in humans to modulate biological processes and treat disorders has proven successful in several applications, such as deep brain stimulation and pacemaker devices. However, conventional bioelectronics are often based on rigid and metallic materials, which can cause a mechanical mismatch, biocompatibility issues, and signal loss when interacting with soft and dynamic biological tissues.^[^ ^1^ ^]^ Conductive polymers (CPs) are a class of bioelectronics that can overcome these limitations by mimicking the properties of biological tissues.^[^ ^2^ ^]^ However, like classical bioelectronics, prefabricated substrate‐bound CP devices are limited by biomechanical mismatch and can cause inflammation, scarring, and loss of function.^[^ ^3^ ^]^ An emerging research field is the in situ assembly of CPs in vivo triggered by enzymes. Thus, several methods have been presented, including expressing polymerization enzymes by genetic engineering,^[^ ^4^ ^]^ utilizing endogenous enzymes,^[^ ^5^ ^]^ and administering enzymes along with the monomers, taking advantage of endogenous metabolites.^[^ ^6^ ^]^ Polymers that self‐assemble into conductive frameworks in vivo were also developed,^[^ ^7^ ^]^ eliminating the need for polymerization catalysts altogether.^[^ ^8^ ^]^ The latter also solved the problem of external connection and could relay external stimulations for neuronal activity and rectify arrhythmias.^[^ ^8^, ^9^ ^]^ Although these in situ techniques enhance biocompatibility and cellular integration, premanufactured substrate‐bound bioelectrodes still surpass their precision in electrode geometry.^[^ ^10^ ^]^ In semiconductor processing, photopatterning is central for fabricating complex structures with precise dimensions and shapes, such as sensors, electronic devices, and energy devices.^[^ ^11^ ^]^ High spatial control down to the nanometer scale has been achieved using optical photolithography.^[^ ^11b^ ^]^ Transferring this technology to in vivo conditions would allow for control of structural details and shape formation, enabling precise interaction with tissues and cells. However, the literature on photopolymerizations directly accessing conjugated polymers is limited.^[^ ^12^ ^]^ Among the reported techniques, several significant limitations arise, particularly when considering in vivo applications. Oxidative photopolymerizations, for instance, are performed in organic solvents and involve oxidants such as CCl_4_,^[^ ^13^ ^]^ diaryliodonium salts,^[^ ^14^ ^]^ and potassium dichromate^[^ ^15^ ^]^ under UV light. Alternatively, reductive photopolymerization, which employs electron‐poor Grignard monomers under visible light, has been explored.^[^ ^16^ ^]^ However, these methodologies are incompatible with in vivo applications. This poses a significant challenge for the in vivo photopolymerization of conjugated polymers. To address this issue, we explored two approaches to oxidative photopolymerization in vivo. First, we used newly discovered thiophene monomers, specifically 3Es (EDOT‐Trimers), to form conjugated polymers directly in vivo. Second, we combined 3Es with the poly(3, 4‐ethylenedioxythiophene)butoxy‐1‐sulfonate (PEDOT‐S) variant known as A5.^[^ ^7^ ^]^ A5 forms nanoparticles in an aqueous solution that aggregates into a temporary conductive hydrogel in vivo by absorbing the ions from the tissue. Thus, photopolymerizing 3Es within the A5‐formed hydrogel would enhance stability solely in light‐exposed regions and create a conductive photo pattern in vivo.

Herein, we report on using photopatterning in vivo to create geometrically controlled conductive structures using zebrafish (*Danio rerio*) and chicken embryos (*Gallus gallus*) as models and the discovery and formulation of materials that enabled this.

## 2 Results and Discussion

### 2.1 Material Characterization and Method Optimization

**Figure 1:**
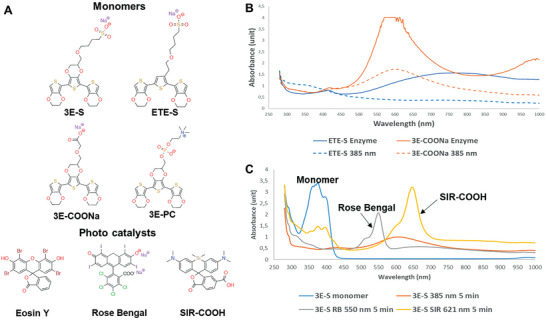
Photopolymerization reaction in solution. A) Chemical structures of thiophene‐based monomers and photocatalysts. B) Comparison of enzymatic polymerization (solid lines) and photopolymerization (dashed lines) for ETE‐S (blue) and 3E‐COONa (red). C) Photopolymerization of 3E‐S (blue line) for 5 min using UV light (red line), Rose Bengal/green light (grey line), and SIR‐COOH/red light (yellow line).

We sought to identify monomers for creating conductive polymers using photopolymerization. Initial tests with the 2,5‐bis(2,3‐dihydrothieno[3,4‐*b*][1,4]dioxin‐5‐yl)thiophene (EDOT‐Thiophene‐EDOT [ETE], **Figure** **1**A) derivatives that we used in previous studies showed no formation of a conductive structure after light exposure of wavelengths covering far‐UV to red (Figure 1B; Figure S1A,B, Supporting Information).

Upon further exploration of potential EDOT derivatives, we pinpointed the sulphonate‐substituted core structure 2,2′,2′‘,3,3′,3′‘‐hexahydro‐5,5′:7′,5′'‐terthieno[3,4‐b][1,4]dioxine (referred to as EDOT‐EDOT(S)‐EDOT [3E‐S]), a compound with a lower oxidation potential compared to the analogous ETE‐S derivative (Figure 1A).^[^ ^17^ ^]^ 3E‐S was resynthesized, together with two newly designed analogs, 3E‐COONa and 3E‐PC (Figure 1A). The 3E and ETE monomers showed a similar effect on cell viability (Figure S3, Supporting Information)^[^ ^8^ ^]^, and we could confirm that the oxidation potential is in general lower for the 3E derivatives compared to ETE derivatives (3E/ETE‐S 0.47 V/0.63 V vs CH_3_CN/TBAHFP (0.1 m),^[^ ^17^ ^]^ 3E/ETE‐COONa 0.10 V/0.30 V versus Ag/AgCl^[^ ^18^ ^]^ and 3E/ETE‐PC 0.05 V/0.35 V versus Ag/AgCl (Figure S2, Supporting Information)). When exposed to far‐UV light (385 nm), 3E monomers (0.4 mg mL^−1^) formed a dark blue‐green solution within 5 min. Analysis showed the same absorption spectrum as for using an enzymatic process (Horse Radish Peroxidase (HRP)/H_2_O_2_), a polymerization process earlier reported, with a distinct broad peak around 600 nm (3E‐COONa, Figure 1B). However, because of limited penetration and tissue damage by UV light, using longer wavelengths is desirable, e.g., green or preferably red light for the near‐infrared window (600–1300 nm) for biological tissues.^[^ ^19^ ^]^ A catalytic amount of photocatalyst Rose Bengal was used to photopolymerize 3E‐S with a green light (550 nm, 65 mW cm^−2^) for 5 min, giving similar results as far‐UV (Figure 1C).

**Figure 2:**
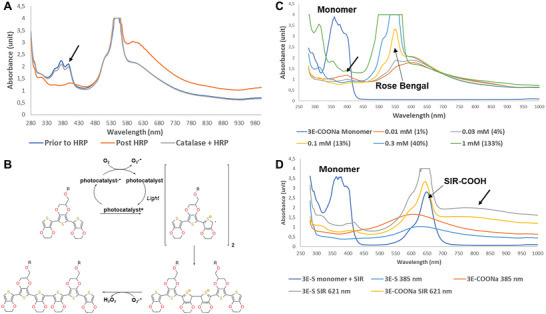
Mechanism and the optimization of photopolymerization reaction in solution. A) Hydrogen peroxide formation in the reaction B) Proposed mechanism of the photopolymerization reaction of 3E monomers using a photocatalyst C) Evaluation of Rose Bengal catalyst loading. D) Analysis of photopolymerization reaction at far‐UV and using SIR‐COOH (red light) after 15 min.

Oxidative photopolymerization of electron‐rich thiophene derivatives has been shown to produce polythiophene products according to a SET mechanism. In addition, reactions using photo catalysts such as Rose Bengal can proceed via a SET mechanism in which oxygen is consumed and hydrogen peroxide is formed.^[^ ^20^ ^]^ To confirm that the photopolymerization of 3Es adheres to a similar mechanism and that hydrogen peroxide is formed, the reaction using 3E‐COONa with Rose Bengal was stopped after 2.5 min at ≈70–80% completion (**Figure** **2**A). No further decrease of the monomer (black arrow in Figure 2A) was detected after the light was turned off. The addition of HRP instantly converted the remaining monomer into the product. However, no additional conversion of the monomer was detected if Catalase, which catalyzes the decomposition of hydrogen peroxide to water and oxygen, was added before HRP. This points to the formation of hydrogen peroxide in the reaction and indicates that the reaction follows the mechanism outlined in Figure 2B and corroborates the previously proposed SET mechanism using photocatalysts.^[^ ^12^, ^20^ ^]^

The short reaction time is crucial for in vivo experiments with zebrafish because of the limited time the fish can be outside the water. A reaction time of 5 min was considered acceptable; however, the key question was how much photocatalyst would be required to achieve a complete reaction within this timeframe. After 5 min, some remaining monomers could be detected using 1% catalyst (peak at 380–400 nm, black arrow), but already at 4% catalyst loading, most of the monomer was converted to product (Figure 2C). The reaction conversion was also dependent on the concentration of the monomer; a 10‐fold increase from 0.4 to 4 mg mL^−1^ significantly reduced the reaction efficiency due to the monomers and emerging polymers absorbing the light. Increasing the surface area relative to the volume circumvented this problem (Figure S1E, Supporting Information).

**Figure 3:**
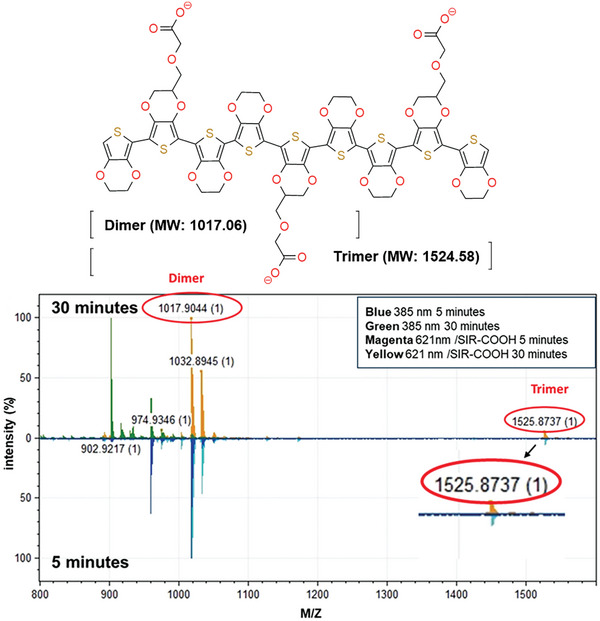
Mass spectrometry analysis of the photopolymerization reaction. The structure of the products and MALDI‐MS for reaction with 3E‐COONa using far‐UV (385 nm), and SIR‐COOH and red light (621 nm) for 5 and 30 min.

SIR‐COOH^[^ ^21^ ^]^ is a recent SI‐analog of fluorescent rhodamine dyes with an excitation maximum in far‐red (650 nm) that has not been used for oxidative photopolymerization. Using SIR‐COOH at 621 nm (red light) gave similar results as with Rose Bengal (green light) and far‐UV but with a slightly lower conversion rate of 3E‐S (Figure 1C) and 3E‐COONa (Figure S1C, Supporting Information). The reaction was completed after 15 min (Figure 2D). However, when using SIR‐COOH and red light, an additional peak/band appeared at 800 nm (Figure 2D, black arrow). Analyses using matrix‐assisted laser desorption/ionization mass spectrometry (MALDI MS) of reaction mixtures from SIR‐COOH and far‐UV conditions at 385 nm showed the formation of dimers in both cases but trimers only in the case of using red light (621 nm) and SIR‐COOH as a photocatalyst (**Figure** **3**; Figure S1F,G, Supporting Information). In addition, the increased absorption after 700 nm represents an oxidated polymer.^[^ ^22^ ^]^ In such case, the red light at 621 nm, combined with the SIR‐COOH, generates larger polymers and oxidizes the polymer not observed previously using Rose Bengal catalyzed reactions. The sequential addition of more monomer to already completed reactions, comprising dimers and trimers, and further exposure to red light increased the peak after 700 nm relative to the one at 600 nm (reduced polymer) (Figure S1D, Supporting Information). Thus, an increased formation of trimers gave more oxidated polymer.

3E‐PC had a slower reaction rate in the photopolymerization but, more importantly, was the least stable of the 3E monomers in an aqueous solution; spontaneous formation of black residues was observed. This led to a focus on 3E‐S and 3E‐COONa.

### 2.2 Optimization in Tissue Mimic Model

**Figure 4:**
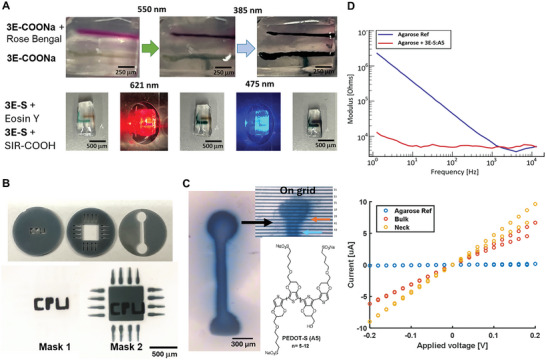
Optimization in tissue mimic model. A) Selective sequential photopolymerization using 3E‐COONa (20 mg mL^−1^ in MilliQ water) with and without photocatalyst Rose Bengal and Eosin Y and SIR‐COOH using 3E‐S (20 mg mL^−1^ in MilliQ water) in an agarose gel cast (0.5%). B) Sequential photopolymerization using 3E‐COONa (20 mg mL^−1^ in MilliQ water) in water and far‐UV (385 nm) on agarose gel cast with photo masks to produce overlay pattern with precise geometric control. C) Geometry controlled formation using photopatterning of conductive bioelectronics on an agarose cast gel using an oxygenated mixture of 3E‐S:A5, Rose Bengal, Urea, PEG‐400, Triton X‐100, and illuminated with green light (20x objective). Evaluated using an array of parallel Au electrode lines (15 µm between lines). D) Electrochemical impedance spectroscopy measurement using a gold‐coated micropipette electrode on agarose gel cast.

**Figure 5:**
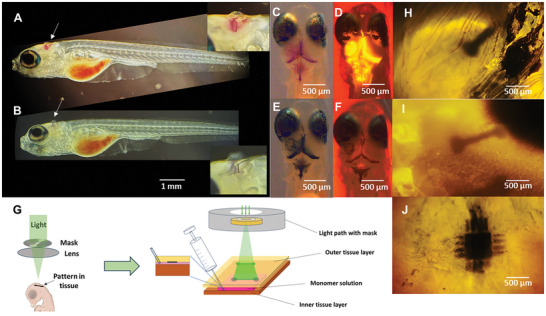
Photopolymerization ex vivo in zebrafish larvae and chicken brain. Microinjection (10 nL) using glass capillary of 3E‐COONa (20 mg mL^−1^ in MilliQ water) with Rose Bengal into the brain ventricle of zebrafish larvae Casper mutants (Tg(elav3:GCaMP6f )) and sagittal imaging before (A) and after (B) photopolymerization using green light (550 nm). Transversal imaging before (C, Brightfield, D), far‐UV 385 nm) and after (E, Brightfield, F), far‐UV 385 nm) photopolymerization. G) Illustration of intermembrane application (created with BioRender.com). H–J) Formation of spatially controlled bioelectronics on the chicken brain using an oxygenated mixture of 3E‐S:A5, Rose Bengal, Urea, PEG‐400, and Triton x‐100 illuminated with green light (20x objective).

Low‐concentration agarose gel cast (0.5%) mimics brain tissue with similar mechanical characteristics and weak visual light absorption and are therefore suitable for evaluating the photopolymerization reaction in vitro.^[^ ^23^ ^]^ Injections of the 3E‐COONa (20 mg mL^−1^) solutions with and without Rose Bengal resulted in partial aggregation with limited diffusion in the agarose gel cast (**Figure** **4**A and visualization of aggregation of 3E‐COONa see Figure S4A, Supporting Information). Only the injection containing Rose Bengal responded to the green light, forming a black residue. Far‐UV exposure for 5 min polymerized the injection, which lacked Rose Bengal. Furthermore, selective polymerization was achieved with the injected 3E‐S containing SIR‐COOH when exposed to red light, compared to 3E‐S containing Eosin Y. Subsequent exposure to blue light polymerized the Eosin Y containing injection (Figure 4A). This highlights the potential for UV/blue/green/red light polymerization and enables the possibility for orthogonal control via photocatalysts and different wavelengths. Spatial control of shape is one of the key advantages of photopolymerization and is currently lacking with current methods in the in situ formation of bioelectronics in vivo.^[^ ^4^, ^5^, ^6^, ^8^ ^]^ 3E‐COONa was applied to the surface of the agarose gel cast and absorbed into the top layer. Photo masks, placed in the Neutral Density (ND) filter holder in the microscope (after the collimator for the light source but before the focusing lenses), were used for photopatterning with far‐UV light (Figure 4B). The mask design was selected to represent different detail levels (geometrical figures and letters) to estimate the resolution of light‐induced patterns. Sequential photomasks with complementary patterns enabled a layer‐by‐layer process where the letters were first formed, followed by the microprocessor pattern with micron‐level resolution (Figure 4B). Some of the applied monomer oiled out as a gel‐like structure in the agarose model, which was also observed in the brain tissue. Another route of administration would be to apply a more water‐soluble monomer (compared to 3E‐COONa) to a surface area of the skin or an organ and let it permeate into the tissue before photopolymerization using a photomask to generate a conductive pattern only in the area of illumination. Thus, to optimize the formulation, it was important to increase the concentration of the monomer and improve the diffusion into and within the tissue. In parallel, to increase the conductivities in the initial vivo experiments (**Figure** **5**), an alternative monomer formulation was developed. The idea was to introduce A5, a variant of the conductive polymer PEDOT‐S that diffuses in tissue.^[^ ^7^ ^]^ Thus, applying a 3E and A5 mixture (3E:A5) followed by photopolymerization of the 3E monomers would generate a polymerized core and crosslink A5 and thereby stabilize the structure and prevent disintegration solely where 3E:A5 preformed hydrogel was exposed to light, whereas the rest of the mixture would diffuse away. Furthermore, using the more water‐soluble 3E‐S instead of 3E‐COONa (Figure S4, Supporting Information), in a formulation of 3 m urea, PEG‐400 (1%), and Triton X‐100 (1%), the concentration of the monomer was increased from 20 to 40 mg mL^−1^ together with A5 (10 mg mL^−1^). In addition, the solution was oxygenated, which improved the photopolymerization process.^[^ ^24^ ^]^ This formulation gave a photopolymerized structure that was more homogenous than noticed before. There was no pattern formation if 3E‐S was excluded from the mixture (Figure S5, Supporting Information). The 3E‐S:A5 was applied to the surface of an agarose gel cast and illuminated for 15 min with green light (550 nm) using a photomask, followed by rinsing the surface and placement on an array of parallel Au electrode lines (Figure 4C). The bone‐shaped pattern was conductive, displaying currents in the microampere range and an expected difference between off (<100 nA) and on the pattern (4–10 µA). In addition, electrochemical impedance spectroscopy with a gold‐coated micropipette electrode contacting the round pattern and using an Ag/AgCl as reference electrode demonstrated a 100‐fold difference between only agarose and agarose with a photopolymerized material (Figure 4D) in the lower frequency region. Further electrochemical characterizations (cyclic voltammetry, output curve (ID vs VDS), and current‐voltage curves) show typical semiconductor characteristics and similar performance after repeated voltage sweeps and, therefore, could be useful for generating OECT devices (Figure S6, Supporting Information).

### 2.3 Evaluation in Ex Vivo Models

The 3E‐COONa together with Rose Bengal were dissolved in water and injected (≈1–10 nL) into sedated zebrafish larvae (Casper mutants [Tg(elav3:GCaMP6f )]) using 30 µm glass capillary (Figure 5A). Illumination using green light (550 nm) for 15 min led to polymerization, observed as a color change from red to black (Figure 5B). The polymerization was corroborated by the depletion of the fluorescence signal from the monomer in far‐UV (Figure 5C–F). Next, we turned to a larger animal to form spatially controlled conductive structures using photopatterning. Similar to humans, layers of membranes cover the chicken brain, and the intermembrane space enables a thin and well‐spread‐out application of the monomer.^[^ ^25^ ^]^ The oxygenated mixture, including 3E:A5, Rose Bengal, urea, PEG‐400, and Triton X‐100, was applied to the intermembrane space of the brain in euthanized chicken embryos (13 embryo development day [EDD]) (Figure 5G). Photopolymerization, as shown in Figure 5H–J, using green light for 15 min and two types of masks, successfully generated patterns of polymers on the brain.

### 2.4 Application In Vivo—Spatial Controlled Formation of Conductive Structures in Zebrafish

**Figure 6:**
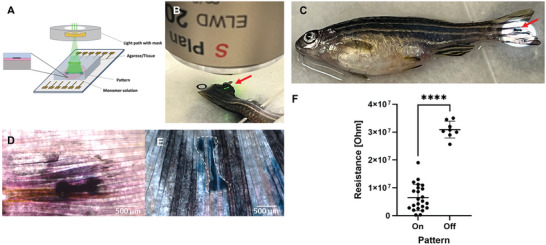
Photopolymerization in vivo in zebrafish caudal fin. A) Illustration of the photopolymerization of zebrafish caudal fin. B) Structured green light illumination of the caudal fin using photo mask for patterning. C) 3D patterned caudal fin. C and D) Formed pattern using 3E‐S:A5, D) after photopolymerization, residues clearly visible as pink coloring from remaining Rose Bengal and black deposits from 3E‐S:A5. E) Formed pattern (white contour) on live fish after 24 h with limited remaining reactants. F) Resistance measurement on the caudal fin of a live fish 24 h post patterning using an array of parallel Au electrode lines (15 µm between lines) at several places (data points in the plot) on and off the pattern with constant distance (15 µm). (*n* = 5 fish, *p* value < 0.0001 in an unpaired *t*‐test).

The zebrafish caudal fin is a highly dynamic model system, and we have previously demonstrated its suitability as a model for the formation and evaluation of conductive bioelectronics.^[^ ^6^, ^8^ ^]^ The adult zebrafish were anesthetized, and the tail fin was carefully spread out and stacked between a glass slide and an agarose cast (1% in Ringer medium). The oxygenated mixture containing 3E:A5, urea, PEG‐400, Triton X‐100, and Rose Bengal was applied between the fin and the glass slide (**Figure** **6**A). The fin was exposed to green light for 15 min using a photo mask (bone structure) in the ND filter holder to generate the pattern (Figure 6B). The zebrafish were then revived and transferred to a fish tank. The pattern was clearly visible, with some residues remaining of the rose bengal and mixture not exposed to the light (Figure 6C,D). The zebrafish (*n* = 5) were monitored for 24 h, and no detrimental effects were observed. Thereafter, zebrafish were anesthetized and positioned in the microscope, showing a pronounced pattern; all residues were cleared (Figure 6E; Figure S7, Supporting Information). The pattern was retained despite physical stress on the structure from 24 h of swimming, which corroborates with the permeation of the mixture into the tissue rather than adhesion to the skin. Transient stability is expected of these materials. We have previously shown stability from days to weeks in vivo, dependent on the application with transient inflammation imminent to the injection, the materials themselves did not sustain or prolong the inflammatory response.^[^ ^8^, ^9^ ^]^ Some inconsistency could be spotted in the pattern, probably due to the different properties of the structures that constitute the fin (fin rays versus inter‐ray mesenchyme) and how well the mixture is absorbed and retained. The resistance on–off the pattern was measured using an array of parallel Au electrode lines (15 µm between lines) using a Keithley sourcemeter (sedated again after 24 h of swimming). The 3D structure of the fin rays is amenable to only contacting adjacent lines. We verified that the pattern was electrically conductive (Figure 6F). This was the first time we could confirm conductivity in the caudal fin model since it has been notoriously difficult to contact the polymeric structure in previous studies and only successful after dehydrating the fin.^[^ ^8^ ^]^ The current magnitude aligns with our previous studies in zebrafish fins and brain slices.^[^ ^6^, ^8^ ^]^

## 3 Conclusion

In this study, we show for the first time the formation of conductive structures in vivo using photopolymerization. We have developed a technique for generating organic bioelectronic structures with precise spatial and temporal control through photopatterning. Rational design and synthesis of 3E monomers have resulted in compounds that enable the formation of conductive structures after photopolymerization and, at the same time, meet the stringent requirements for in vivo use. Balancing photopolymerization capability with solubility and stability presents a challenge and must be considered; our optimized monomer formulations have increased concentration and diffusion rates, meeting diverse application needs. Furthermore, the photopolymerization of the monomer 3E stabilized also an otherwise unstable aggregation of the PEDOT‐S derivative A5. The effectiveness of these monomers has been validated in our model systems, including adult, larval zebrafish, and chicken embryos, paving the way for future advancements in advanced in situ‐generated bioelectronics.

## 4 Experimental Section

### Animal Ethics

This study was conducted according to Swedish national legislation and European Community guidelines for animal studies. All procedures were approved by the ethical committee in Malmö‐Lund (5.8.18‐05993/2018 and 5.8.18‐05748/2022). All experimental procedures involving chicken embryos were completed before the 14th day of embryonic development (EDD 14), a stage which, according to the regulatory guidelines, does not categorize them within the scope of animal experimentation.

### Chemicals

All chemicals were purchased from commercial suppliers and were used as received. Rose Bengal (95%) was purchased from Sigma Aldrich (330000), and SIR‐COOH was purchased from SpiroChrome (SC004). Microtiter plates, 96‐well black with transparent bottom (Greiner 655096), were used. Agarose LE, from Analytical Grade, Promega Corporation was used. TritonX‐100 (X100), Urea (U631), PEG400 (P3265) were purchased from Sigma Aldrich.

### Ringer Solution

116 mm NaCl (Sigma–Aldrich M7506), 2.9 mm KCl (Sigma–Aldrich P5405), 1.8 mm CaCl2 (Sigma–Aldrich P5405), 5 mm HEPES (Sigma–Aldrich H3375), adjusted to pH 7.2 using NaOH (1 m) (Sigma–Aldrich S5881) or HCl (1 m) (Applichem A0659).

### Surfactant Solution

Triton‐X 100 (10 µL) and Polyethylenglycol 400 (10 µL) were added to Urea (180 mg) dissolved Ringer's solution (1 mL), resulting in a 3 m Urea 1% Triton‐X 100, 1% PEG in Ringer solution.

### 3E‐S:A5 Solution

Rose Bengal (1 uL,10 mm, Sigma Aldrich 330000) in MilliQ water was added to 3E‐S (0.8 mg) dissolved in the surfactant solution (20 uL). The solution was oxygenated by bubbling oxygen through the solution. The oxygenated 3E‐S solution was added to a vial containing A5 (0.2 mg), which gave a final concentration of 3E‐S (40 mg mL^−1^), 3E‐S, A5 (10 mg mL^−1^), and Rose Bengal (0.4 mm). The solution was freshly prepared for each experiment and immediately used after preparation.

### Oxidation Potential ETE‐COONa, 3E‐COONa, ETE‐PC, and 3E‐PC

Oxidation potentials were determined using a Keithley sourcemeter 2612B (Keithley Instruments) with 20 uL 3E monomer [10mg mL^−1^] in NaCl (7 mm) and Au/AgCl electrodes. The sweep speed was 5 mV s^−1^.

### Cell Viability for 3E Monomers Using an MTT Assay

Cell toxicities were determined according to Hjort et al. using HLF‐1 cells.^[^ ^8^ ^]^ NOTE The values above 0.1 mg mL^−1^ were difficult to determine correctly due to the precipitation of polymerized 3E monomers after a few hours, which led to a gradually reduced concentration over time.

### Photopolymerization in Microtiter Plates

A solution of monomer (2 µL, 20 mg mL^−1^ in MilliQ water), optional photocatalyst (1 µL, 10 mm in MilliQ water for RoseBengal and DMSO for SIR‐COOH), and MilliQ water were added to a black clear bottom 96‐well microtiter plate to get a total volume of 100 µL. The solution was illuminated by light (UV 385 nm, Green 550 nm, or Red 621 nm, D‐LEDI Nikon (65 mW cm^−2^ Newport Powermeter 843‐R) for a specified time. The absorbance spectrum (280–1000 nm) was then recorded (Tecan SparkCyto 400).

### Catalyst Loading

Performed according to the general method photopolymerization in microtiter plates using 3E‐COONa (20 mg mL^−1^ in MilliQ water) at 1, 4, 13, 40, and 113 mol% of Rose Bengal (10 mm in DMSO).

### Matrix‐Assisted Laser Desorption Ionization Mass Spectra (MALDI MS)

MALDI MS was obtained on MALDI‐TOF/TOF Autoflex Speed (Bruker Daltonics) using external calibration with a peptide calibration standard. Samples were prepared as previously described (Chem. Mater. 2022, 34, 2752−2763). Briefly, samples were dissolved in 0.1% TFA at a concentration of 2 mg mL^−1^. and the MALDI matrix 2, 5‐dihydroxybenzoic (DHB) acid was prepared at 10 mg mL^−1^ in 50:50 [v/v] methanol/0.1% TFA in water. The sample solution (0.5 µL) was air‐dried on the flat surface of a stainless‐steel plate. Next, 0.5 µL of DHB was deposited over the sample layer, and the mixture was allowed to dry. The spectra were recorded in reflectron‐positive mode.

### Generation of Masks for Photolithography

The CAD files for the patterning masks were created using the 3D modeling Web Tool Tinkercad (Autodesk). The masks were printed using 3D Printing UV‐sensitive Resin (Type “Basic”, Anycubic) on a Monoprice MP Mini SLA LCD Resin 3D printer (Monoprice).

### Photopolymerization Using Agarose Gel Cast—Wavelength Specific Photopolymerization

1) Green and UV Light

A monomer solution (2 µL, formulated using 15 µL E3‐COONa (20 mg mL^−1^ in MilliQ water) with and without 1 µL Rose Bengal (10 mm in DMSO)) was injected as two parallel lines into an agarose mold (0.5% agarose in Ringer solution) using a Hamilton syringe. The agarose mold was transferred to the microscope setup (Nikon ECLIPSE FN1) which was then illuminated by green light (550 nm) for 5 min followed by UV light (385 nm) for 5 min using a 4x/0.10 Nikon objective. Imaging was performed with the same objective.

2) Red and Blue Light

A monomer solution (2 µL, formulated using 15 µL E3‐COONa (20 mg mL^−1^ in MilliQ water) with and 1 µL Rose Bengal (10 mM in DMSO)) or 1 µL Eosin Y (10 mM in DMSO)) was injected as two parallel lines into an agarose mold (0.5% agarose in Ringer solution) using a Hamilton syringe. The agarose mold was transferred to the microscope setup (Nikon ECLIPSE FN1) which was then illuminated by red light (621 nm) for 5 min followed by blue light (475 nm) for 5 min using a 4x/0.10 Nikon objective. Imaging was performed with the same objective.

### Photopolymerization Using Agarose Gel Cast—Spatial Controlled Photopolymerization

3E‐COONa (5 µL, 20 mg mL^−1^ in MilliQ water) was added to the surface of an agarose cast (0.5% in Ringer solution) and the solution was dried in forming a thin layer of the monomer. Two photo masks were inserted into the light path (ND filter slots after the collimator of the light source but before focusing lenses. The agarose cast was transferred to the microscope setup (Nikon ECLIPSE FN1) which was then illuminated by UV light (385 nm) for 5 min with Mask 1 followed by Mask 2 for 5 min using a 20x/0.45 Nikon objective. Imaging was performed using a 4x/0.10 Nikon objective.

### Photopolymerization Using Agarose Gel Cast—Patterning and Electric Measurements

3E‐S:A5 solution (2 µL, see preparation of 3E‐S:A5 solution) was added to the surface of a glass slide. A 1×2×0.5 cm agarose mold was placed on top of the monomer solution (0.5% agarose in Ringer solution). The photo mask was inserted into the light path (ND filter slots) after the collimator of the light source but before focusing lenses. The agarose mold was transferred to the microscope setup (Nikon ECLIPSE FN1) which was then illuminated by Green light (561 nm) for 15 min using a 20x/0.45 Nikon objective. Imaging was performed using a 4x/0.10 and a 20x/0.45 Nikon objective. The patterned agarose was removed from the glass slide and washed with 3 mL MilliQ water.

The agarose mold was then placed on interdigitated Au electrodes connected to a Keithley sourcemeter 2612B (Keithley Instruments). The pattern was facing toward the Au electrodes. Two of the interdigitated electrodes were contacted using external microelectrodes. An applied voltage was swept, and the resulting current was registered. This was repeated for all interdigitated electrode leads covering the conductive polymer. The distance between adjacent electrodes was 15 µm, and the width was 2.5 mm.

### Electrochemical Characterization—Impedance Analysis

A Gamry 1010B potentiostat was utilized for obtaining EIS measurements. A gold‐covered micropipette was utilized as a working electrode probed inside the agarose gel with and without diffused photopolymerized (15 min green light) 3E‐S:A5 with Rose Bengal (5 µL, see preparation of see preparation of 3E‐S:A5 solution), and an Ag/AgCl as reference electrode in Ringer solution. Python 3.9 and Origin were implemented for the data analysis and the data was fitted with a Randle´s circuit R (R||C).

### Electrochemical Characterization—Cyclic Voltammetry, Output Curves and Current‐Voltage Curves

3E‐S:A5 5 µL containing Rose Bengal was applied to an array of parallel Au electrode lines and then exposed to 15 min of green light. For cyclic voltammetry three cycles between −0.8 and 0.6 V with 0.05 V steps were performed and the scan rate was 50 mv s^−1^. For output curves, the drain voltage was swept from 0 to −0.6 V with 0.1 V steps and the gate voltage from −0.4 to 0.5 V with 0.1 V steps. The material was scanned 10 times for the current‐voltage curves. A Keithley 2612B with custom LabVIEW software was utilized for the acquisition of the OECT characteristics. A Gamry 1010B potentiostat was utilized for obtaining EIS measurements. Python 3.9 and Origin were implemented for the data analysis. The OECT characterization to determine was performed in Ringer solution. For EIS the channel electrodes were shorted and were utilized as a working electrode. The data was fitted with a Randle´s circuit R (R||C)

### Photopolymerization Ex Vivo—Zebrafish Larvae

The monomer solution contained 15 µL E3‐COONa (20 mg mL^−1^ in MilliQ water) and 1 µL Rose Bengal (10 mm in DMSO)

Before microinjection and photopolymerization, larva (Casper mutant (Tg(elav3:GCaMP6f)) on nacre background) were terminally sedated with tricaine (ethyl 3‐aminobenzoate methanesulfonate; 0.2 mg mL^−1^) for a minimum of 10 min until movements had ceased and the fish did not respond to vibrations caused by tapping close to the tricaine container. The larva was placed on its side on a plate filled with 1% agarose (Agarose, LE, Analytical Grade, Promega Corporation) in an E3 medium that had been allowed to solidify. The plate was then transferred, to the microinjection setup, and a capillary with a 30 mm diameter tip (cat. No. BM100T‐15. Bevelled, straight, shortened + firepolished ends from Biomedical‐Instruments GMBH) filled with the monomer solution was inserted into the ventricle. The total injection volume was estimated to be <10 nL. After injection, the larva was transferred to the microscope setup (Nikon ECLIPSE FN1), and the head was illuminated by green light for 15 min using a 4x/0.10 Nikon objective. Photopolymerization was confirmed by imaging using brightfield light and UV (385 nm) with the same objective.

### Photopolymerization Ex Vivo—Ex Vivo Patterning Procedures were Performed on Excised Brain–Dura Complexes

Brain samples with an intact dura mater were isolated from 13‐day‐old chicken embryos. After euthanizing the embryos by decapitation, the brain and the dura mater were excised together using a modified protocol according to (https://doi.org/10.3791/200144‐v).  Brain‐dura complexes were stored in Ringer solution at 8 °C.

For patterning, 2 µL A5‐3E‐S solution (prepared as described in section “A5‐3E‐S solution”) was injected into the subdural space on top of the interhemispheric space of brain**–**dura complexes using a 10 µL Microliter Syringe (Hamilton Company). Injected samples were placed on a plate filled with 1% agarose (Agarose, LE, Analytical Grade, Promega Corporation) in Ringer’ solution that solidified with the injection site orientated ventrally. The plate was then transferred to the photopolymerization setup, and a patterning mask was inserted into the light path/ND‐filter slot of the microscope after the collimator of the light source but before focusing lenses. The pattern was focused on the surface of the sample and irradiated with 550 nm light for 15 min. Imaging was performed using a 4x/0.10 Nikon objective.

### Photopolymerization In Vivo—In Vivo–Tail Fin Light Patterning

Adult zebrafish (Danio rerio) AB wildtype were used for the patterning experiment.

Before the patterning procedure, zebrafish were anesthetized with tricaine medium (0.2 mg mL^−1^) until opercular movements had ceased and the fish did not respond to vibrations caused by tapping close to the tricaine container. The anesthetized fish was placed on its side on 1% agarose in Ringer's solution. A piece of moist tissue paper was placed over the fish, exposing the caudal fin to keep the body from drying. The caudal peduncle was carefully lifted with tweezers, and a microscope cover glass plate (24×60 mm, No. 1) was placed under the caudal fin. The caudal fin was dried with a paper towel. Freshly prepared 3E‐S–A‐5 mixture (3 µL) was extruded between the tail fin and the glass slide. The glass slide was transferred to the photopolymerization setup, and a patterning mask was inserted into the light path (ND‐filter slot) after the collimator of the light source but before focusing lenses. The pattern was focused on the surface of the sample and irradiated with 550 nm light for 15 min. After patterning, excess material was washed off the tail fin. Zebrafish were revived directly by flushing the gills with fresh aquarium water and transferred to an aquarium for observation.

### Photopolymerization In Vivo—Electronic Evaluation of A5:3E‐S Caudal Fin Pattern

The conductivity of the A5:3E‐S pattern in vivo was measured 24 h after the patterning procedure. After 24 h, fish were anesthetized as described previously, the polymer pattern on the tail fin was placed facing an array of parallel Au electrode lines connected to a Keithley sourcemeter 2612B (Keithley Instruments). Two of the electrode lines were contacted using external microelectrodes. An applied voltage was swept (−0.2 to 0.2 V), and the resulting current was registered. This was repeated for all interdigitated electrode leads covering the conductive polymer. The distance between adjacent electrodes was 15 µm, and the width was 2.5 mm. Statistical analysis using an unpaired *t*‐test was performed in GraphPad Prism 10.

## Conflict of Interest

The authors declare no conflict of interest.

## Author Contributions

F.E., T.A., and M.S. contributed equally to this work. F.E. and R.O. performed methodology; R.O., T.A., A.H.M., and M.A.S. performed Design and synthesis; F.E. and M.S., performed Spectroscopical characterization; K.H., P.E., S.B., and F.E. performed Zebrafish experiments; S.B. and F.E. performed Chicken experiments; A.S.Y. performed Cell work; L.H.B. and G.M.V. performed Mass spectrometry; M.H., S.B., X.S., T.A., M.S., J.Y.G., and F.E. performed Electrical characterization and analysis; M.B., M.H., D.T.S. and R.O. acquired funds; F.E. and R.O. supervised the project; F.E. and R.O. wrote the original draft; All authors reviewed and edited the draft, wrote, reviewed and edited the final manuscript.

## Supporting information



Supporting Information

## Data Availability

The data that support the findings of this study are available in the supplementary material of this article.

## References

[1] R. Chen , A. Canales , P. Anikeeva , Nat Rev Mat. 2017, 2, 16093. 10.1038/natrevmats.2016.93 PMC6707077 31448131

[2] a) C. Boehler , S. Carli , L. Fadiga , T. Stieglitz , M. Asplund , Nat. Protoc. 2020, 15, 3557; 33077918 10.1038/s41596-020-0389-2

[3] a) J. Rivnay , H. Wang , L. Fenno , K. Deisseroth , G. G. Malliaras , Sci. Adv. 2017, 3, e1601649; 28630894 10.1126/sciadv.1601649 PMC5466371

[4] J. Liu , Y. S. Kim , C. E. Richardson , A. Tom , C. Ramakrishnan , F. Birey , T. Katsumata , S. Chen , C. Wang , X. Wang , L.‐M. Joubert , Y. Jiang , H. Wang , L. E. Fenno , J. B.‐H. Tok , S. P. Pașca , K. Shen , Z. Bao , K. Deisseroth , Science. 2020, 367, 1372. 32193327 10.1126/science.aay4866 PMC7527276

[5] G. Tommasini , G. Dufil , F. Fardella , X. Strakosas , E. Fergola , T. Abrahamsson , D. Bliman , R. Olsson , M. Berggren , A. Tino , E. Stavrinidou , C. Tortiglione , Bioactive Materials. 2022, 10, 107. 34901533 10.1016/j.bioactmat.2021.08.025 PMC8637319

[6] X. Strakosas , H. Biesmans , T. Abrahamsson , K. Hellman , M. S. Ejneby , M. J. Donahue , P. Ekström , F. Ek , M. Savvakis , M. Hjort , D. Bliman , M. Linares , C. Lindholm , E. Stavrinidou , J. Y. Gerasimov , D. T. Simon , R. Olsson , M. Berggren , Science. 2023, 379, 795. 36821679 10.1126/science.adc9998

[7] A. H. Mousa , D. Bliman , L. H. Betancourt , K. Hellman , P. Ekström , M. Savvakis , X. Strakosas , G. Marko‐Varga , M. Berggren , M. Hjort , F. Ek , R. Olsson , Chem. Mater. 2022, 34, 2752. 35360437 10.1021/acs.chemmater.1c04342 PMC8944941

[8] M. Hjort , A. H. Mousa , D. Bliman , M. A. Shameem , K. Hellman , A. S. Yadav , P. Ekström , F. Ek , R. Olsson , Nat. Commun. 2023, 14, 4453. 37488105 10.1038/s41467-023-40175-3 PMC10366153

[9] U. Aydemir , A. H. Mousa , C. Dicko , X. Strakosas , M. A. Shameem , K. Hellman , A. S. Yadav , P. Ekström , D. Hughes , F. Ek , M. Berggren , A. Arner , M. Hjort , R. Olsson , Nat. Commun. 2024, 15, 6774. 39117721 10.1038/s41467-024-51111-4 PMC11310494

[10] F. Tian , J. Yu , W. Wang , D. Zhao , J. Cao , Q. Zhao , F. Wang , H. Yang , Z. Wu , J. Xu , B. Lu , J. Colloid Interface Sci. 2023, 638, 339. 36746052 10.1016/j.jcis.2023.01.146

[11] a) D. Zhang , C. Li , G. Zhang , J. Tian , Z. Liu , Acc. Chem. Res. 2024, 57, 625; 10.1021/acs.accounts.3c00750 38295316

[12] E. F. Woods , A. J. Berl , J. A. Kalow , Chem. Photo. Chem. 2021, 5, 4.

[13] T. Iyoda , M. Kitano , T. Shimidzu , J. Chem. Soc., Chem. Commun. 1991, 1618.

[14] Y. Yagci , F. Yilmaz , S. Kiralp , L. Toppare , Macromol. Chem. Phys. 2005, 206, 1178.

[15] S. A. Piletsky , E. V. Piletska , K. Karim , F. Davis , S. P. J. Higson , A. P. F. Turner , Chem. Commun. 2004, 2222. 10.1039/b408387c 15467882

[16] E. F. Woods , A. J. Berl , J. A. Kalow , Angew. Chem., Int. Ed. 2020, 59, 6062. 10.1002/anie.201915819 31922643

[17] D. Mantione , E. Istif , G. Dufil , L. Vallan , D. Parker , C. Brochon , E. Cloutet , G. Hadziioannou , M. Berggren , E. Stavrinidou , E. Pavlopoulou , ACS Appl. Electron. Mater. 2020, 2, 4065.

[18] T. Abrahamsson , M. Savvakis , F. Ek , B. Burtscher , D. Byun , M. J. Donahue , D. Hughes , C. Musumeci , E. Rulander , M. Hjort , J. Y. Berggren , D. T. Simon , R. Olsson , X. Strakosas , Soon to be submitted, 2024.

[19] R. Weissleder , Nat. Biotechnol. 2001, 19, 316. 11283581 10.1038/86684

[20] A. Srivastava , P. K. Singh , A. Ali , P. P. Singh , V. Srivastava , RSC Adv. 2020, 10, 39495. 35515398 10.1039/d0ra07400d PMC9057485

[21] a) G. Lukinavičius , L. Reymond , E. D'Este , A. Masharina , F. Göttfert , H. Ta , A. Güther , M. Fournier , S. Rizzo , H. Waldmann , C. Blaukopf , C. Sommer , D. W. Gerlich , H.‐D. Arndt , S. W. Hell , K. Johnsson , Nat. Methods. 2014, 11, 731; 24859753 10.1038/nmeth.2972

[22] a) D. G. Harman , R. Gorkin , L. Stevens , B. Thompson , K. Wagner , B. Weng , J. H. Chung , M. in het Panhuis , G. G. Wallace , Acta Biomater. 2015, 14, 33; 25484333 10.1016/j.actbio.2014.11.049

[23] R. Pomfret , G. Miranpuri , K. Sillay , Ann Neurosci. 2013, 20, 118. 25206029 10.5214/ans.0972.7531.200309 PMC4117117

[24] E. Mitraka , M. J. Jafari , M. Vagin , X. Liu , M. Fahlman , T. Ederth , M. Berggren , M. P. Jonsson , X. Crispin , J. Mater. Chem. A Mater. 2017, 5, 4404. 28580144 10.1039/c6ta10521a PMC5436492

[25] a) K. Dasgupta , J. Jeong , Genesis. 2019, 57, e23288; 30801905 10.1002/dvg.23288 PMC6520190

[26] G. Hu , T. Emrick , J. Am. Chem. Soc. 2016, 138, 1828. 26841336 10.1021/jacs.5b13156

