# Supplementary material for: In Vivo Photopolymerization: Achieving Detailed Conducting Patterns for Bioelectronics
Source: Adv Sci (Weinh). 2024 Nov 7;11(48):2408628. doi: 10.1002/advs.202408628 (PMC11672244; doi:10.1002/advs.202408628)
Supplement: Supplementary file 1 — Supporting Information [file ADVS-11-2408628-s001.pdf]

ADVANCED SCIENCE
Open Access

Supporting Information

for *Adv. Sci.*, DOI 10.1002/advs.202408628

In Vivo Photopolymerization: Achieving Detailed Conducting Patterns for Bioelectronics

*Fredrik Ek, Tobias Abrahamsson, Marios Savvakis, Stefan Bormann, Abdelrazek H. Mousa, Muhammad Anwar Shameem, Karin Hellman, Amit Singh Yadav, Lazaro Hiram Betancourt, Peter Ekström, Jennifer Y. Gerasimov, Daniel T. Simon, György Marko-Varga, Martin Hjort, Magnus Berggren, Xenofon Strakosas and Roger Olsson**

Supplementary materials for

In Vivo Photopolymerization:

Achieving Detailed Conducting Patterns for Bioelectronics

Authors: Fredrik Ek[1#], Tobias Abrahamsson[2#], Marios Savvakis[2#], Stefan Bormann[1], Abdelrazek H. Mousa[3], Muhammad Anwar Shameem[3], Karin Hellman[1], Amit Singh Yadav[1], Lazaro Hiram Betancourt[4], Peter Ekström[1], Jennifer Gerasimov[2], Daniel Simon[2], György Marko-Varga[4], Martin Hjort[1], Magnus Berggren[2], Xenofon Strakosas[2] and Roger Olsson[1,3]*

Affiliations:

[1]Chemical Biology and Therapeutics, Department of Experimental Medical Science, Lund University, SE-221 84 Lund, Sweden,

[2] Laboratory of Organic Electronics, Department of Science and Technology, Linköping University, 601 74 Norrköping, Sweden.

[3]Department of Chemistry and Molecular Biology, University of Gothenburg, SE-405 30 Gothenburg, Sweden.

[4] Division of Oncology, Department of Clinical Sciences Lund, Lund University, Lund, Sweden

*Corresponding author. Roger Olsson Email: roger.olsson@med.lu.se

[#]Authors contributed equally

Table of Content Material and Methods

1. **Chemicals**
2. **Synthesis of 3E monomers**
3. **Supplementary figures**

   S1 Photopolymerization

   S2 Oxidation potentials of 3E monomers

   S3 Cell viability after exposure to 3E monomers

   S4 Solubility of 3E-COONa and 3E-S

   S5 Photopolymerization without 3E-S

   S6 Electrochemical characterization

   S7 Patterning of caudal fin

4. **NMR spectra**

1. Chemicals

3E-PC

3,4-Ethylenedioxythiophene, (2,3-dihydrothieno[3,4-b][1,4]dioxin-2-yl (Hydroxymethyl EDOT), 2-Chloro-1,3,2-dioxaphospholane 2-oxide, Triethylamine, 2-Isopropoxy-4,4,5,5-tetramethyl-1,3,2-dioxaborolane, K_2CO_3 (potassium Carbonate), $NaHCO_3$, NBS, 2-isopropoxy-4,4,5,5'-tetramethyl-l,3,2-dioxaborolane, 2.5 M n-BuLi, PEPPSI-iPr, Na_2SO_4, deuterated NMR solvents ad NMR tubes all were purchased from Sigma Aldrich. Trimethyl amine (2M in acetonitrile) was purchased from TCI. Solvents: DMF, THF, Toluene, MeOH, and Acetonitrile were purchased from VWR and dried on a PureSolvMD5 (inert) solvent purification system.

3E-COONa

The synthesis of 2,3-dihydrothieno[3,4-b][1,4]dioxin-5-yl)-4,4,5,5- tetramethyl-1,3,2-dioxaborolane (step b) has previously been reported. Column chromatography purifications were carried out with a Biotage Select system, utilizing prepacked (Sfär Duo) Silica and C18 silica columns, and samples were wet, dry, or manually dry loaded onto silica and C18 silica Samplets®. High-performance liquid chromatography-mass spectrometry (HPLC-MS) analysis was performed on a Waters system with 2 x 515 HPLC pump, 2424 Evaporative Light Scattering (ELS) Detector, 2998 Photodiode Array (PDA) Detector, SQ Detector 2 (single quadrupole mass detector, ESI ionization), equipped with a XBridge BEH C18 Column (3.5 µm x 4.6 mm x 50 mm, pore size 130 Å). Mobile phase gradient A (water): 95 % H_2O, acetonitrile 5 % with 10 mM NH_4OAc and phase B (organic): 10 % H_2O, 90% acetonitrile, 10 mM NH_4OAc were used in 30–100% or a 10–100% (organic phase B in water phase A), 1.5 mL/min, with injection volume 20 µL. ^{1}H- and ^{13}C-NMR were recorded (at 25 °C) with a Varian Inova-500 Shielded (Oxford AS500) spectrometer at 500 and 126 MHz respectively. ^{1}H-NMR chemical shifts were referenced against the solvent's residual peaks at 7.26 ppm for chloroform-*d* and 3.31 ppm for methanol-d_4. ^{13}C-NMR chemical shifts were referenced against solvent residual peaks at 77.16 ppm for chloroform-*d* and 49.00 ppm for methanol-d_4. All NMR data processing and analysis were performed with MestReNova version 12.04 software. 3,4-Ethylenedioxythiophene and its structural analogues are prone to oxidation, polymerization, and aggregation and should be handled carefully. Substances should be handled carefully at elevated temperatures, high concentrations, and under reduced pressure, in acidic and alkaline conditions. Should be kept dark and stored cold.

<u>2. Synthesis of 3E monomers</u>

3.1 ETE-S[8] and 3E-S[17] were synthesized according to literature procedures.

3.2 3E-COONa

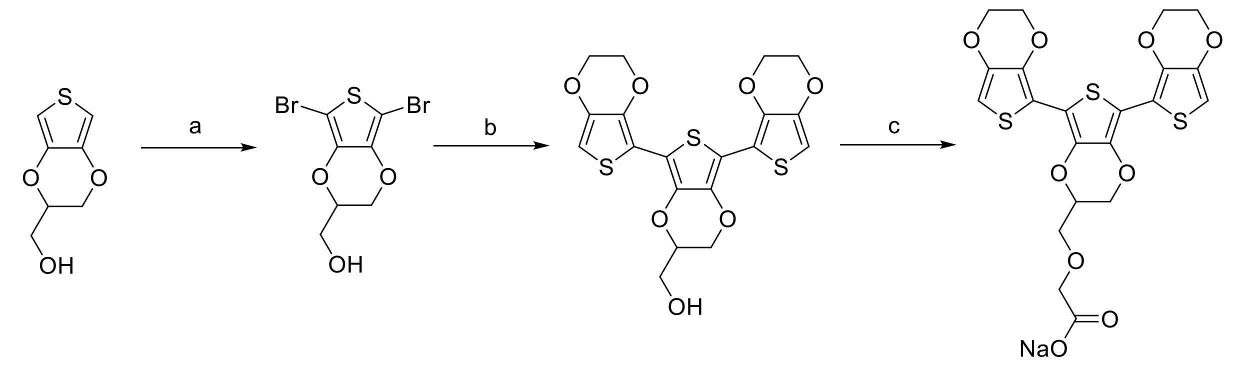

1 **2** **3**

Scheme S1. Synthesis of E3-COONa (**3**). (a) N-bromosuccinimide (2.14 eq.) in DMF, r.t., 24h, 95 %. (b) 2,3-dihydrothieno[3,4-b][1,4]dioxin-5-yl)-4,4,5,5- tetramethyl-1,3,2-dioxaborolane (2.05 eq.), PEPPSI-iPr (5 mol %), 1 M Na_2CO_3 (aq., 2.09 eq.) in THF, 80 °C, 16h, 64 %. (c) i) NaH (1.45 eq.), methyl bromoacetate (1.39 eq.) in THF, 60 °C, 18h. ii) 1 M NaOH (aq., 4.53 eq.) in THF, 1h, 23 %.

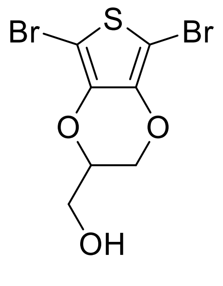

5,7-dibromo(2,3-dihydrothieno[3,4-b][1,4]dioxin-2-yl)methanol (**1**)

A solution of N-bromosuccinimide (5167 mg, 28.74 mmol) in DMF (19 mL) was added dropwise under nitrogen gas flow to a solution of (2,3-dihydrothieno[3,4-b][1,4]dioxin-3-yl)methanol (2386 mg, 13.44 mmol) in DMF (19 mL) in an ice bath. The reaction was stirred for 24h at ambient temperature in the dark and thereafter poured into an ice-cold saturated $NaHCO_3$ (aq.) (45 mL) and stirred for 10 min. The aqueous mixture was diluted with water (100 mL) and extracted with diethyl ether (3 x 45 mL). The combined organic phases were washed with saturated NaHCO3 (aq.) (45 mL), water (3 x 45 mL), and brine (45 mL), dried with MgSO4, and filtered. The organic phase was removed under reduced pressure to yield an almost dry brown oil (yield ~95 %). ^{1}H NMR (500 MHz, Chloroform-*d*) δ 4.33 (dd, J = 11.7, 2.2 Hz, 1H), 4.31 – 4.24 (m, 1H), 4.16 (dd, J = 11.7, 8.0 Hz, 1H), 3.94 (dt, J = 11.8, 4.6 Hz, 1H), 3.87 (dt, J = 12.1, 5.2 Hz, 1H). ^{13}C NMR (126 MHz, Chloroform-*d*) δ 139.59, 139.53, 85.76, 85.73, 74.73, 66.19, 61.23. MS (ESI) *m/z*: $[M + H]^+$: 331.15; $[M - H]^-$: 329.05; calculated for $C_7H_6Br_2O_3S$: 329.99. UV (PDA): λ_{max} (H_2O/acetonitrile): 251.08 nm.

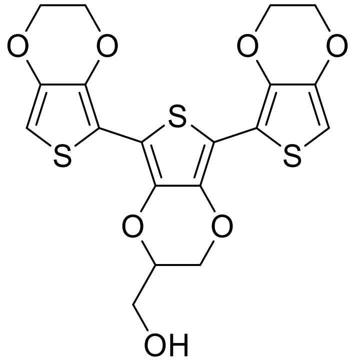

(2,2’,2”,3,3’,3”-hexahydro-[5,5’:7’,5”-terthieno[3,4-b][1,4]dioxin]-2’-yl)methanol (**2**)

PEPPSI-iPr (327 mg, 0.48 mmol, 5 mol%) was added, followed by 1M Na_2CO_3 (20.06 mL, 2.09 eq.) to a solution of 5,7-dibromo(2,3-dihydrothieno[3,4-b][1,4]dioxin-2-yl)methanol (**1**) (3168 mg, 9.60 mmol) and 2,3-dihydrothieno[3,4-b][1,4]dioxin-5-yl)-4,4,5,5- tetramethyl-1,3,2-dioxaborolane (5277 mg, 19.68 mmol, 2.05 eq.) in degassed THF (100 mL). The reaction was heated to 80 °C for 16h under nitrogen gas while kept in the dark. The reaction solution was cooled to room temperature, and the organic phase removed under reduced pressure. THF was added to the residue, and the solution was filtrated through a small silica plug, and dry-loaded onto silica after removal of the organic phase under reduced pressure to a 100 g silica column. The residue was purified by column chromatography using a gradient of EtOAc in heptane (12 to 100%) to give an off-yellow solid (2079 mg, 64 % yield). ^{1}H NMR (500 MHz, Chloroform-*d*) δ 6.26 (d, J = 2.8 Hz, 2H), 4.44 – 4.37 (m, 2H), 4.37 – 4.30 (m, 4H), 4.25 – 4.18 (m, 5H), 3.97 – 3.83 (m, 2H). ^{13}C NMR (126 MHz, Chloroform-*d*) δ 141.48, 141.36, 137.00, 136.94, 136.29, 136.19, 110.08, 110.06, 108.49, 108.15, 97.86, 97.74, 75.16, 74.60, 65.95, 65.20, 65.19, 64.71, 61.44. MS (ESI) *m/z* $[M + H]^+$: 453.34; $[M - H]^-$: 451.14; calculated for $C_{19}H_{16}O_7S_3$: 452.51. UV (PDA): λ_{max} (H_2O/acetonitrile): 375.08(, 386.08 & 355.08) nm.

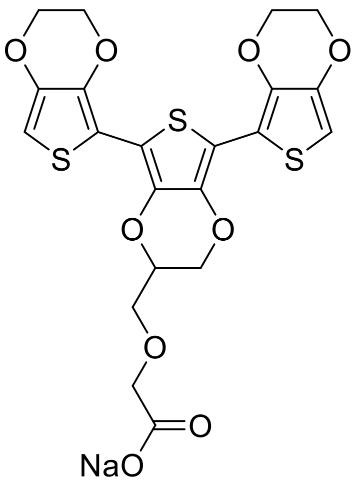

Sodium 2-((2,2',2",3,3',3"-hexahydro-[5,5':7',5"-terthieno[3,4-b][1,4]dioxin]-2'-yl)methoxy)acetate (**E3-COONa**, **3**)

NaH (90%, 171 mg, 6.413 mmol, 1.45 eq.) added was added to a solution of (2,2',2",3,3',3"-hexahydro-[5,5':7',5"-terthieno[3,4-b][1,4]dioxin]-2'-yl)methanol (**2**) (1999 mg, 4.418 mmol) in dry THF (20 mL) at ambient temperature and left to stir for 30 min under a nitrogen atmosphere, followed by addition of methyl bromoacetate (6.143 mmol, 0.6 mL, 1.39 eq.). The reaction was heated at 60 °C for 20h under a nitrogen atmosphere in the dark. The reaction mixture was cooled to ambient temperature, and 1M NaOH (20 mL) was added dropwise and thereafter stirred for 45 min, resulting in a clear dark brown solution. The reaction solution was diluted with water (50 mL) and washed with ethyl acetate (50 mL). The organic phase was extracted with water (50 mL). The combined aqueous phases were washed with ethyl acetate (100 mL), made acidic by dropwise addition of 3 M HCl, and extracted with ethyl acetate (3 x 200 mL). The combined organic phases were washed with water (2 x 250 mL) and brine (200 mL), dried with MgSO4, filtered, and the organic phase removed under reduced pressure. The remaining residue was dissolved in THF (6.5 mL), 1M NaOH (5 mL) was added to reach neutral pH and stirred for 1h. The solvents were

removed under reduced pressure and dry-loaded onto C18 silica and applied on a C18 silica (30g) column. The residue was purified by column chromatography using a acetonitrile gradient in water (10 to 80%) to give the product and after co-evaporation of the aqueous mixture with methanol. The product was dissolved in minimal methanol (30 mL) aided by gentle heating, filtered through cotton, and precipitated in ice-cold diethyl ether (6 x 40 mL) fractions. The fractions were centrifuged at 4000 rpm for 3 min, decanted the diethyl ether and the collected solid product was dried under high vacuum (544 mg, 23% yield). ^{1}H NMR (500 MHz, Methanol-d_4) δ 6.30 (s, 2H), 4.55 – 4.40 (m, 2H), 4.32 (d, J = 3.9 Hz, 4H), 4.26 – 4.14 (m, 5H), 4.01 (s, 2H), 3.88 (dd, J = 10.8, 4.6 Hz, 1H), 3.78 (dd, J = 10.7, 5.9 Hz, 1H). ^{13}C NMR (126 MHz, Methanol-d_4) δ 177.92, 142.69, 142.67, 138.07, 138.04, 137.63, 137.58, 111.23, 111.20, 109.23, 109.21, 98.61, 98.50, 74.79, 72.47, 70.35, 67.71, 66.32, 65.85. HPLC-MS (ESI) m/z [M+H]$^+$: 511.36; [M]$^-$: 509.16; calculated for $C_{21}H_{17}O_9S_3^-$: 509.54. UV (PDA): λ_{max} (H_2O/acetonitrile): 376.08(, 397.08 & 356.08) nm.

3.3 3E-PC

NMR spectra were measured on a Bruker Avance NEO 600MHz violet spectrometer Swedish NMR center at the University of Gothenburg. Column purifications were performed on the Biotag Select flash chromatography system using Biotag Sfär prepacked column.

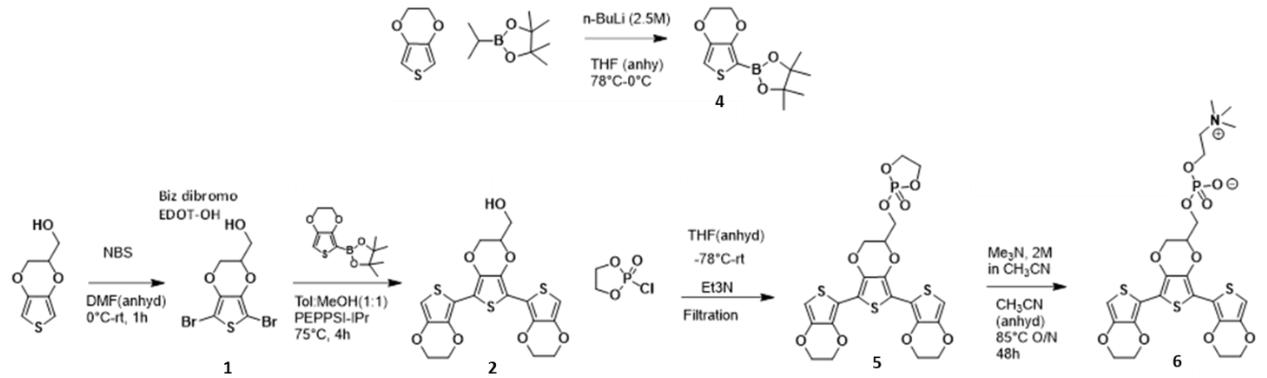

Scheme S2: Synthetic scheme of 3E-PC.

Synthesis of EDOT boronic acid pinacol ester (4)

The synthesis was performed according to the reported literature procedure with slight modifications.(*17*) 3,4-ethylene-dioxythiophene (5.340 g, 37.559 mmol) and 80 mL dry THF were added to a three-necked oven-dried Schlenk flask under inert conditions (N_2). The solution was cooled down to -78°C in an acetone/dry ice bath before adding n-BuLi (2.5M in n-hexane) dropwise. The acetone/dry ice bath was replaced with an ice bath, and the transparent reaction mixture was stirred at 0 °C for 20 min. the resultant yellow orangish solution was cooled down to -78°C in the acetone/dry ice bath, followed by a dropwise addition of 2-isopropoxy-4,4,5,5-tetramethyl-1,3,2-dioxaborolane and the resultant mixture was allowed to warm to ambient temperature slowly and stirred overnight. Upon completion, the reaction mixture was placed in an ice bath and quenched with ice-cold saturated aq. NH_4Cl (85 mL) solution. The resultant suspension was transferred into a 1000 ml separatory funnel and extracted with DEE (3x180 ml). Organic phases were combined, washed with D.I. water (180 mL) and brine (180 mL), and dried

over Na_2SO_4. The solvent was removed at reduce pressure, which gave **4** as a clear, thick, slightly yellowish oil. The crude product was used in the next step without further purification.

^{1}HNMR ($CDCl_3$, δ=ppm, 600MHz): 6.61 (1H, S), 4.28-4.27 (2H. m), 4.16-4.15 (2H, m), 1.32 (12H, broad S).

Synthesis of 3E phosphocholine (3E-PC) (6)

The synthesis was performed according to the reported literature procedure with slight modifications and under strict dark conditions.[26]

Step-1: Trimethylamine (116.5 µL, 1.2 eq) was added to a solution of **2** (315 mg, 0.696 mmol, 1.0 Equiv.) in anhydrous THF (8 ml). The resultant mixture was cooled down to -75 °C in a dry ice acetone bath, 2-chloro-1,3,2-dioxaphospholane 2-oxide (66.6 µL, 0.731 mmol, 1.05 eq) was added dropwise over 5 min and the reaction mixture stirred for 10 min. The cooling bath removed, and the mixture was stirred overnight at room temperature. The reaction was monitored by TLC (eluent: pure ethyl acetate). Upon completion, the reaction mixture (brownish-white suspension) was filtered through a glass fritz, and the brownish filtrate was concentrated under reduced pressure to obtain a brown sticky oil. The intermediate 3E-Oxophospholane (**5**) was found to be highly unstable at ambient conditions and under light and the crude product was directly used in the next step.

Step-2: Trimethylamine in CH_3CN (5.2 ml, 15eq 2M, 13% in CH_3CN) was added to **5** (0.688 g) dissolved in 3 ml dry acetonitrile in a pressure-vial (20 ml, pre-dried in the oven overnight under N_2) at 0°C under inert condition (N_2). The pressure tube was sealed tightly, completely wrapped with aluminum foil and stirred at 85°C for 48 hours. The tightly sealed pressure tube was then cooled to 0°C in an ice bath, and the brownish crude and white precipitation were transferred into a 100 ml flask concentrated under reduced pressure. The crude product (brown sticky/gummy-like material) was triturated with acetonitrile which gave 0.707 g (93%) of **6** as an orange-red solid.

^{1}HNMR (DMSO-d6, δ=ppm, 600MHz): 6.56-6.55 (2H, broad d), 4.48-4.47 (2H, m), 4.35-4.33 (4H, m), 4.24-4.22 (4H, m), 4.19-4.16 (1H, q), 4.07 (2H, broad S), 3.95-3.91 (1H, m), 3.87-3.83 (1H, m), 3.49-3.51 (2H, m), 3.12 (9H, broad S).

^{13}C NMR (DMSO-d6, δ=ppm, 600MHz): 141.07-141.07 (d, J = 6.0 Hz), 136.81-136.72 (d, J = 13.5 Hz), 136.18-136.07 (d, J = 12.1 Hz), 118, 108.89-108.79 (d, J = 15.1 Hz), 106.96-106.84 (d, J = 18.1 Hz), 97.98, 73.38-73.34 (d, J = 6.0 Hz), 66.10, 65.50, 65.10, 64.27, 62.39, 58.32, 53.10, 44.13.

^{31}P-{H}- NMR (DMSO-d6, δ=ppm, 242.9MHz): -1.64

Note: It is necessary to store the 3Es (solid material) at -80 °C. And aqueous solutions need to be protected from light and used within hours of preparation.

3. <u>Supplementary figures</u>

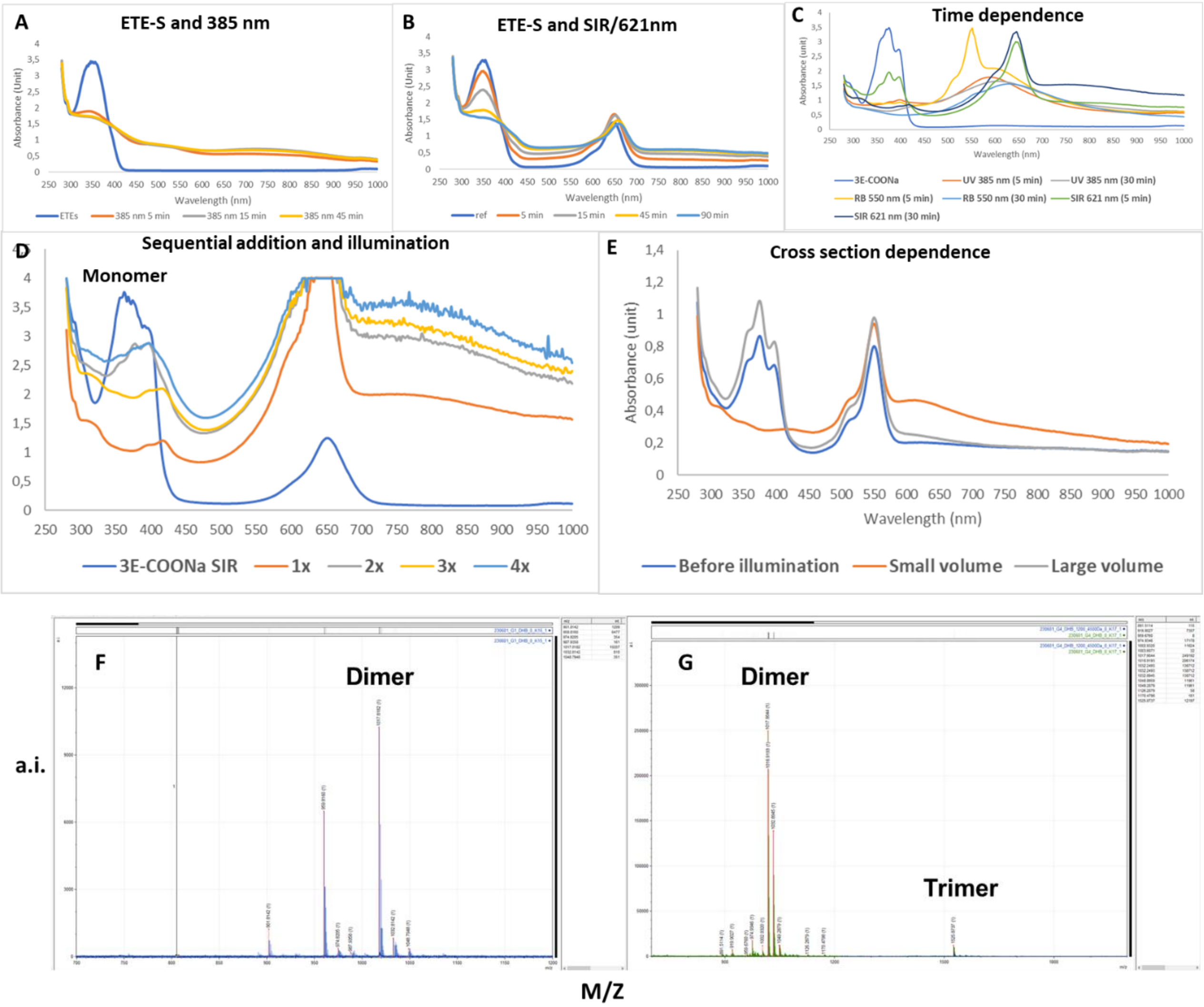

Figure S1. Attempted photopolymerization of ETE-S with UV (A) and with photocatalyst SIR and red light (621 nm) (B). (C) Photopolymerization over time under UV light (385 nm), green light (550 nm) with Rose Bengal and red light (621 nm) with SIR-COOH. (D) Sequential addition and illumination confirm formation of trimers when using SIR-COOH and red light. (E) Cross section dependence in the photo polymerization reaction (after 5 minutes illumination). MALDI-MS for reaction with 3E-COONa using (F) far-UV (385 nm), and (G) SIR-COOH and red light (621 nm) for 5 minutes.

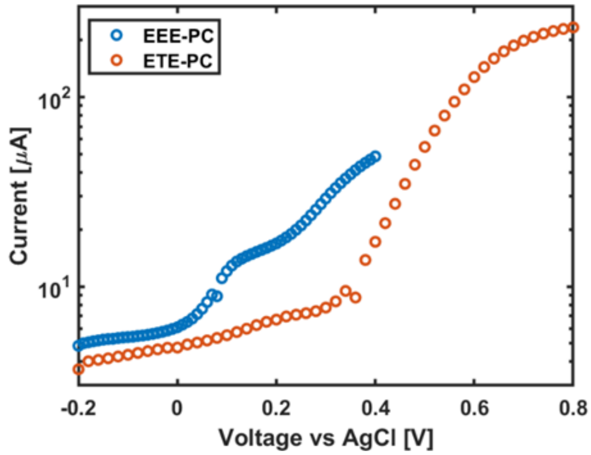

Figure S2. Oxidation potential of ETE-PC and 3E-PC

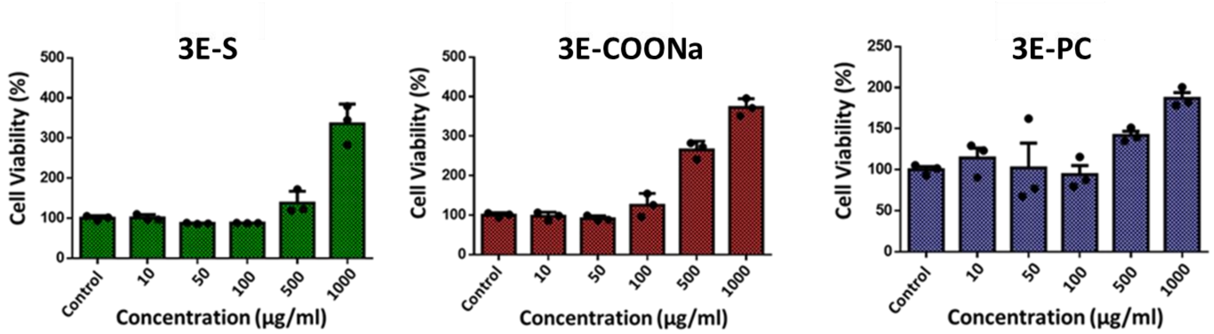

Figure S3. 3E monomers effect on cell viability.

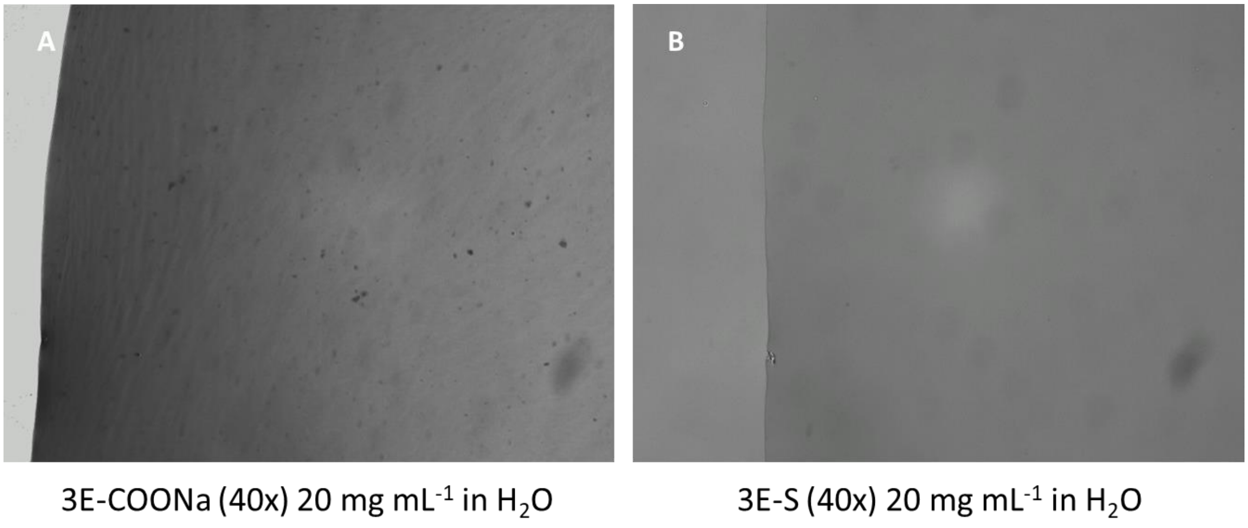

Figure S4. Solubility of 3E-COONa (left) and 3E-S (right) in MilliQ water at 20 mg mL^{-1}.

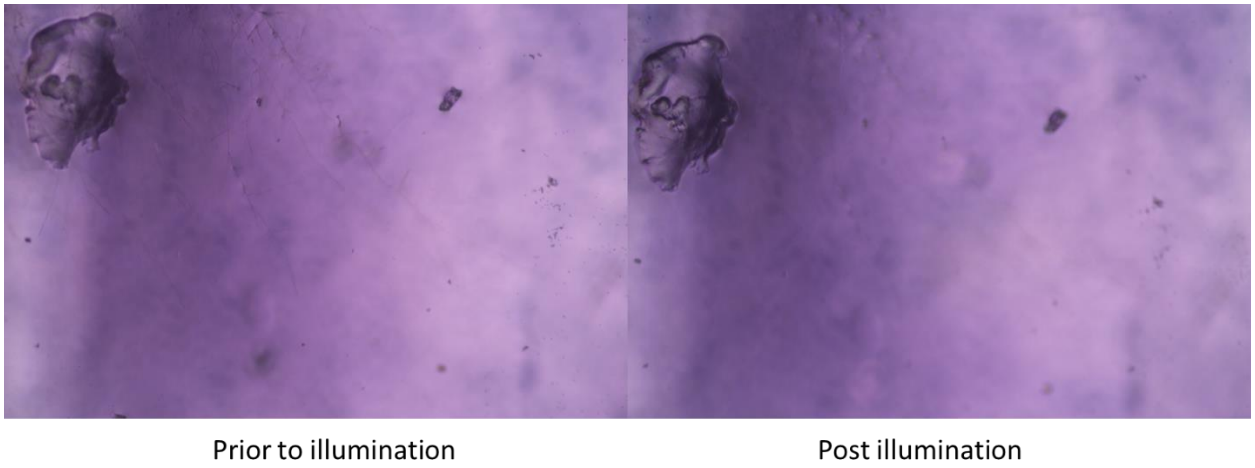

Figure S5. Photopolymerization on an agarose gel cast without 3E-S (A5, Rose Bengal, Urea, PEG-400, Triton X-100) for 15 minutes using green light (20 x objective with photo mask) (A5, Rose Bengal, Urea, PEG-400, Triton X-100)

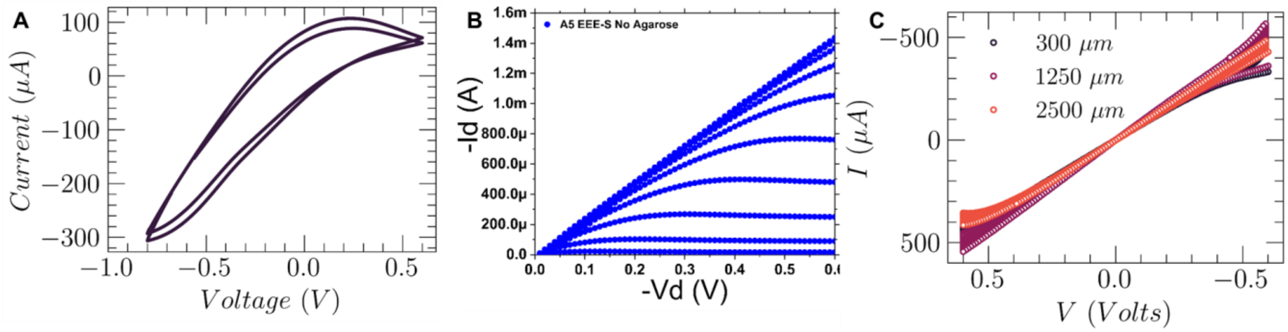

Figure S6. Electrochemical characterization of 3E-S:A5 mixture with Rose Bengal after photopolymerization neat on an array of parallel Au electrode lines (15 µm between lines) using a Keithley sourcemeter (A) Cyclic voltammetry. Three cycles between -0.8V to 0.6V with 0.05 V steps and the scan rate was 50 mv/s. (B) Output curve (ID vs. VDS). For output curves the drain voltage was swept from 0 to -0.6V with 0.1 V steps and gate voltage from -0.4 to 0.5 V with 0.1V steps. (C) Current-voltage curves (n = 10).

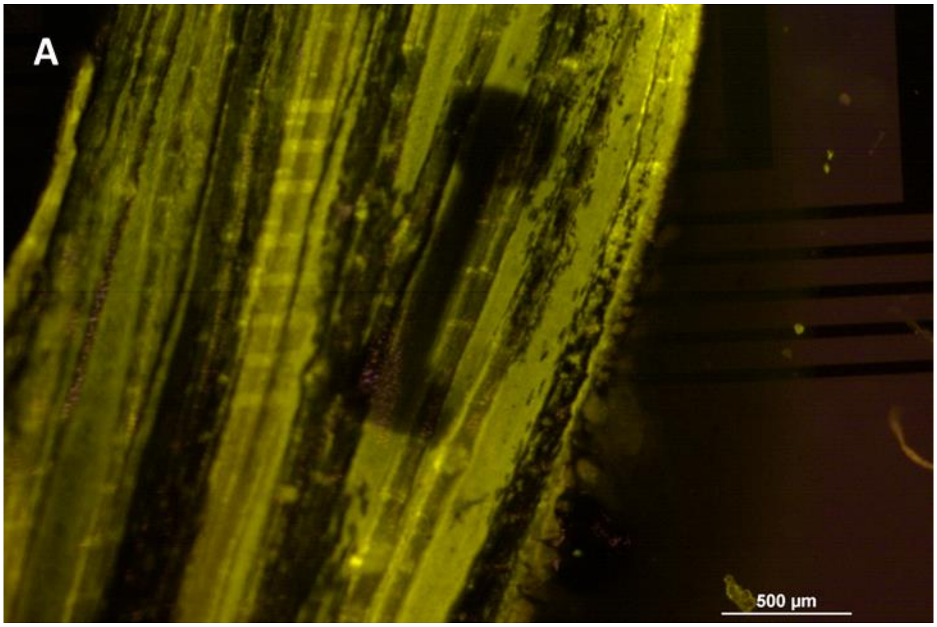

Figure S6. (Left) Patterning of caudal fin using 3E:A5 mixture illuminated by blue light.

4. NMR Spectra

3E-COONa

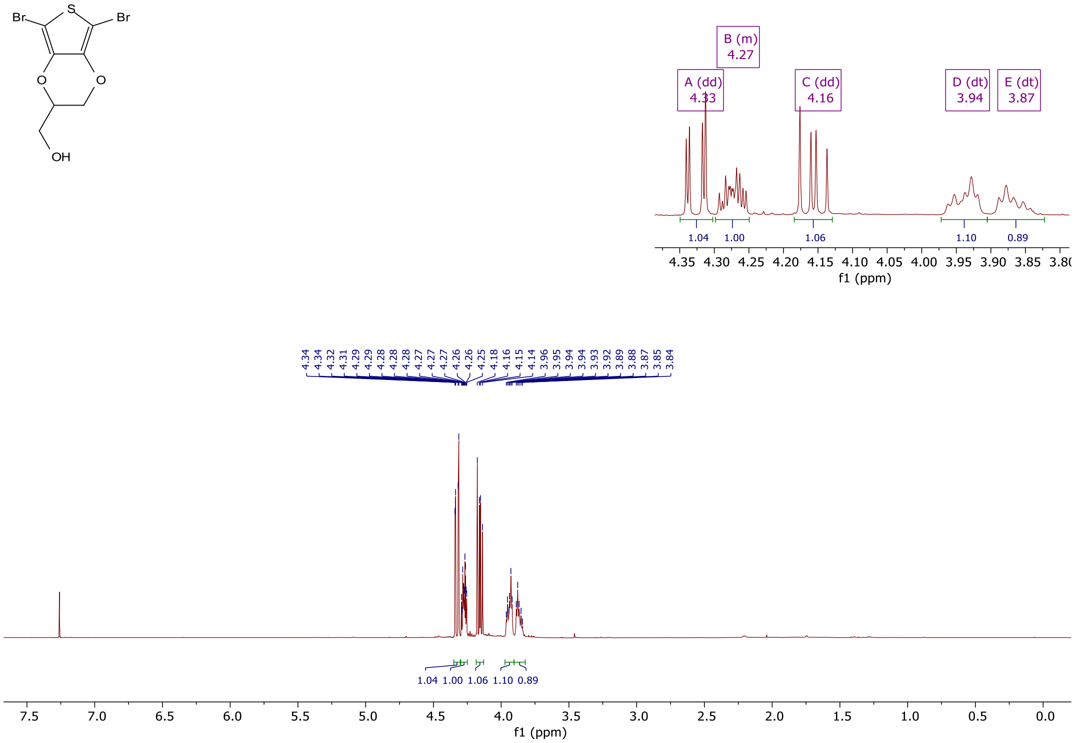

Figure S6. ^{1}H-NMR (chloroform-*d*) spectrum of 5,7-dibromo(2,3-dihydrothieno[3,4-b][1,4]dioxin-2-yl)methanol (**1**).

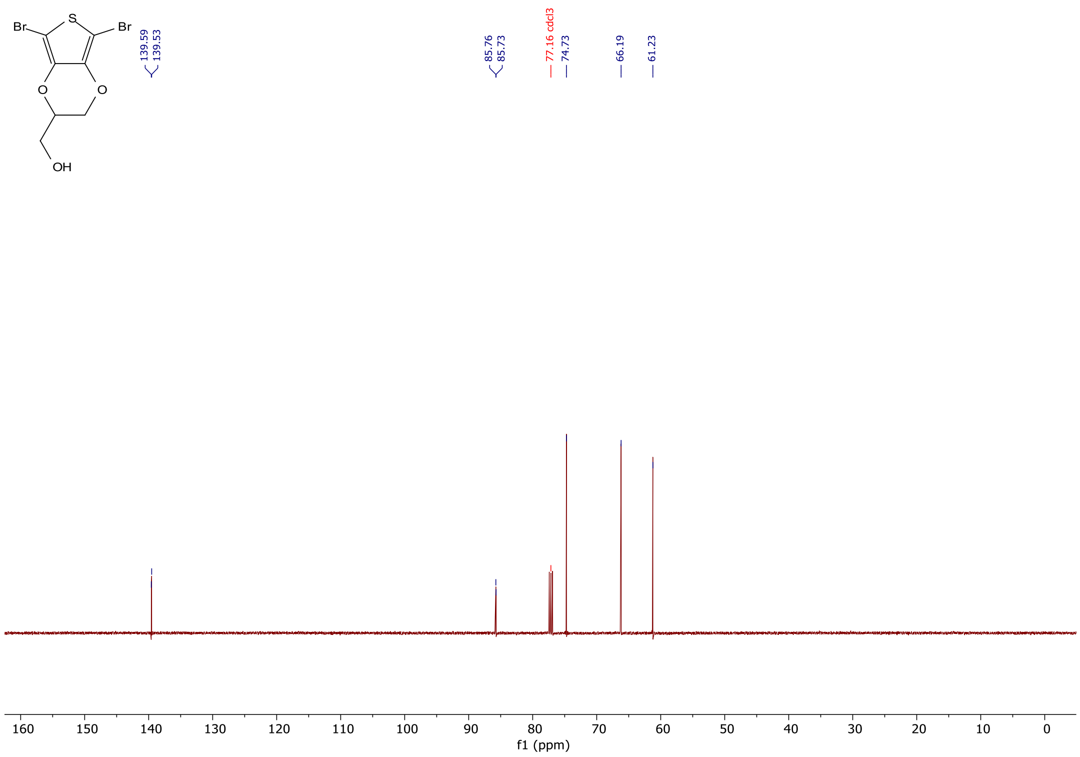

Figure S7. ^{13}C-NMR (chloroform-*d*) spectrum of 5,7-dibromo(2,3-dihydrothieno[3,4-b][1,4]dioxin-2-yl)methanol (**1**).

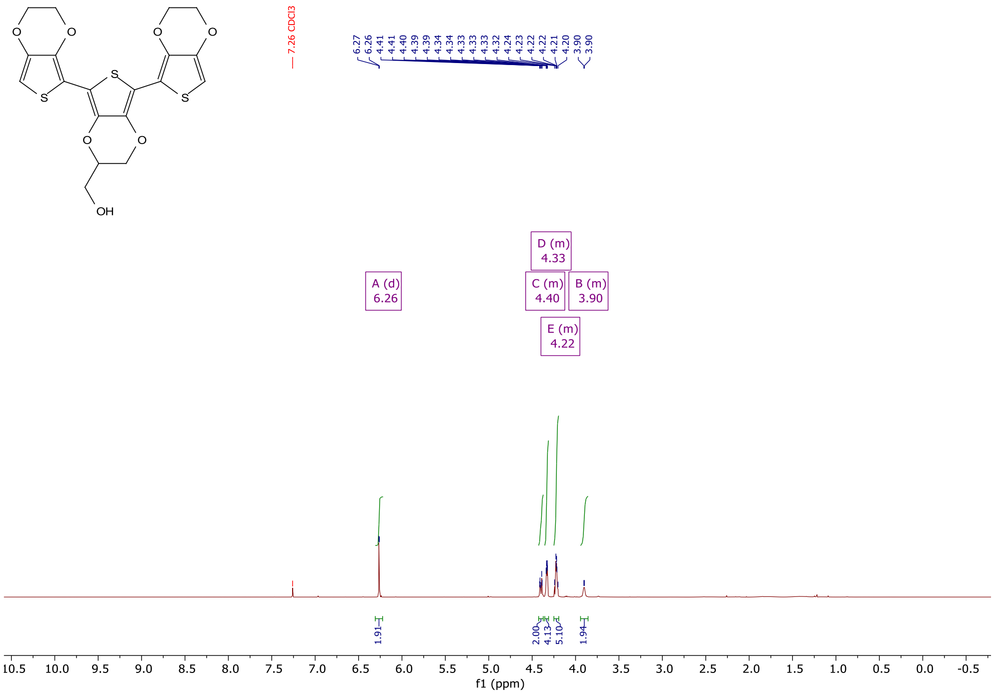

Figure S8. ^{1}H-NMR (chloroform-*d*) spectrum of (2,2',2'',3,3',3''-hexahydro-[5,5':7',5''-terthieno[3,4-b][1,4]dioxin]-2'-yl)methanol (**2**).

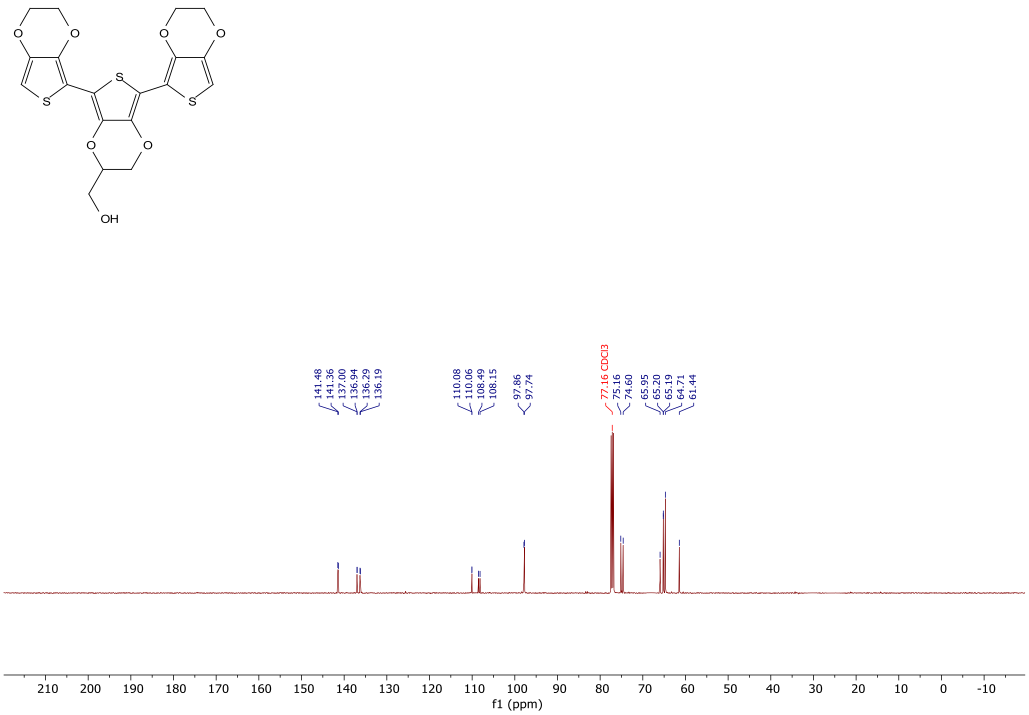

Figure S9. ^{13}C-NMR (chloroform-*d*) spectrum of (2,2',2'',3,3',3''-hexahydro-[5,5':7',5''-terthieno[3,4-b][1,4]dioxin]-2'-yl)methanol (**2**).

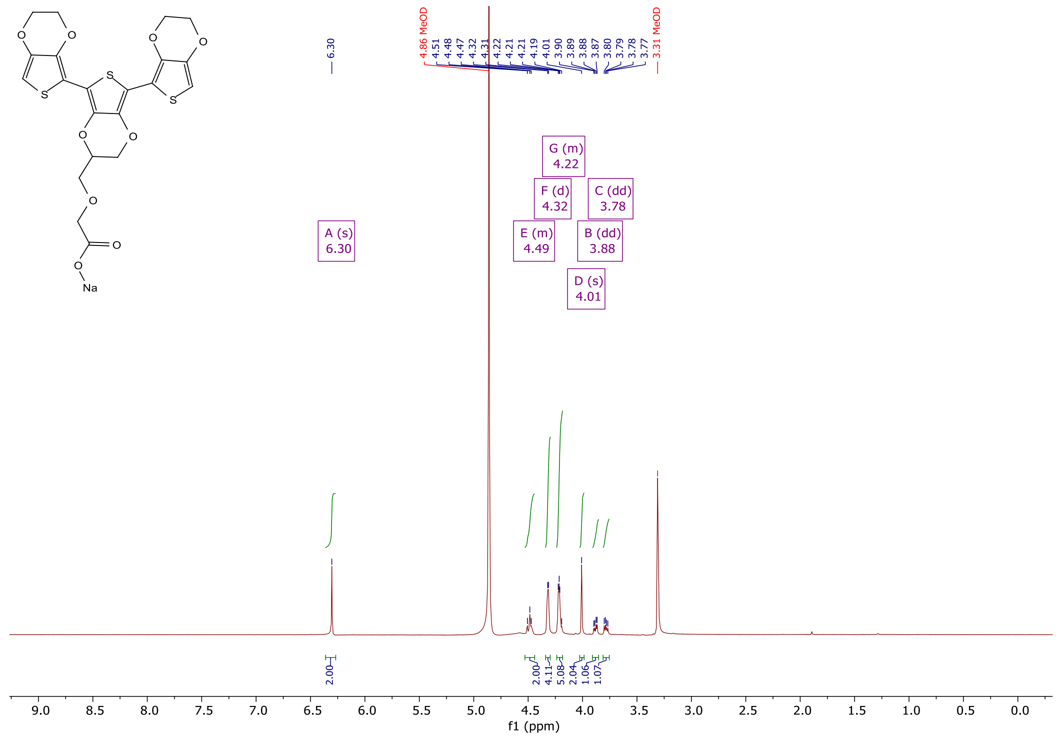

Figure S10. ^{1}H-NMR (methanol-d_4) spectrum of Sodium 2-((2,2’,2”,3,3’,3”-hexahydro-[5,5’:7’,5”-terthieno[3,4-b][1,4]dioxin]-2’-yl)methoxy)acetate (E3-COONa, **3**).

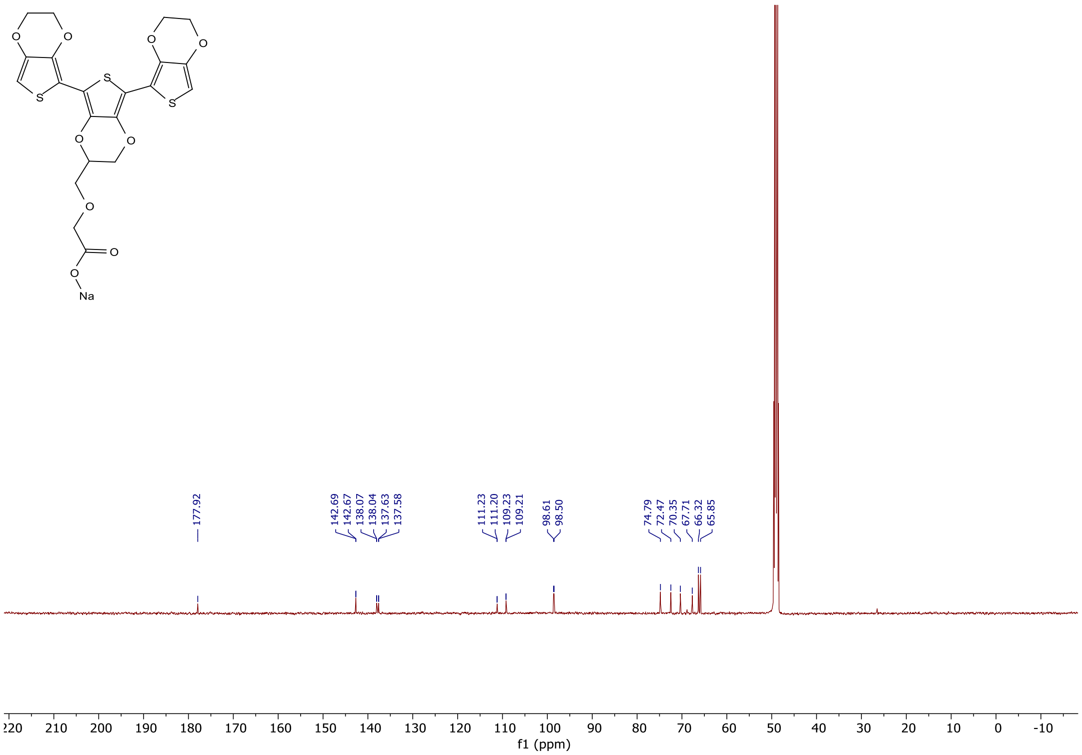

Figure S11. ^{13}C-NMR (methanol-d_4) spectrum of Sodium 2-((2,2',2'',3,3',3''-hexahydro-[5,5':7',5''-terthieno[3,4-b][1,4]dioxin]-2'-yl)methoxy)acetate (E3-COONa, **3**).

3E-PC

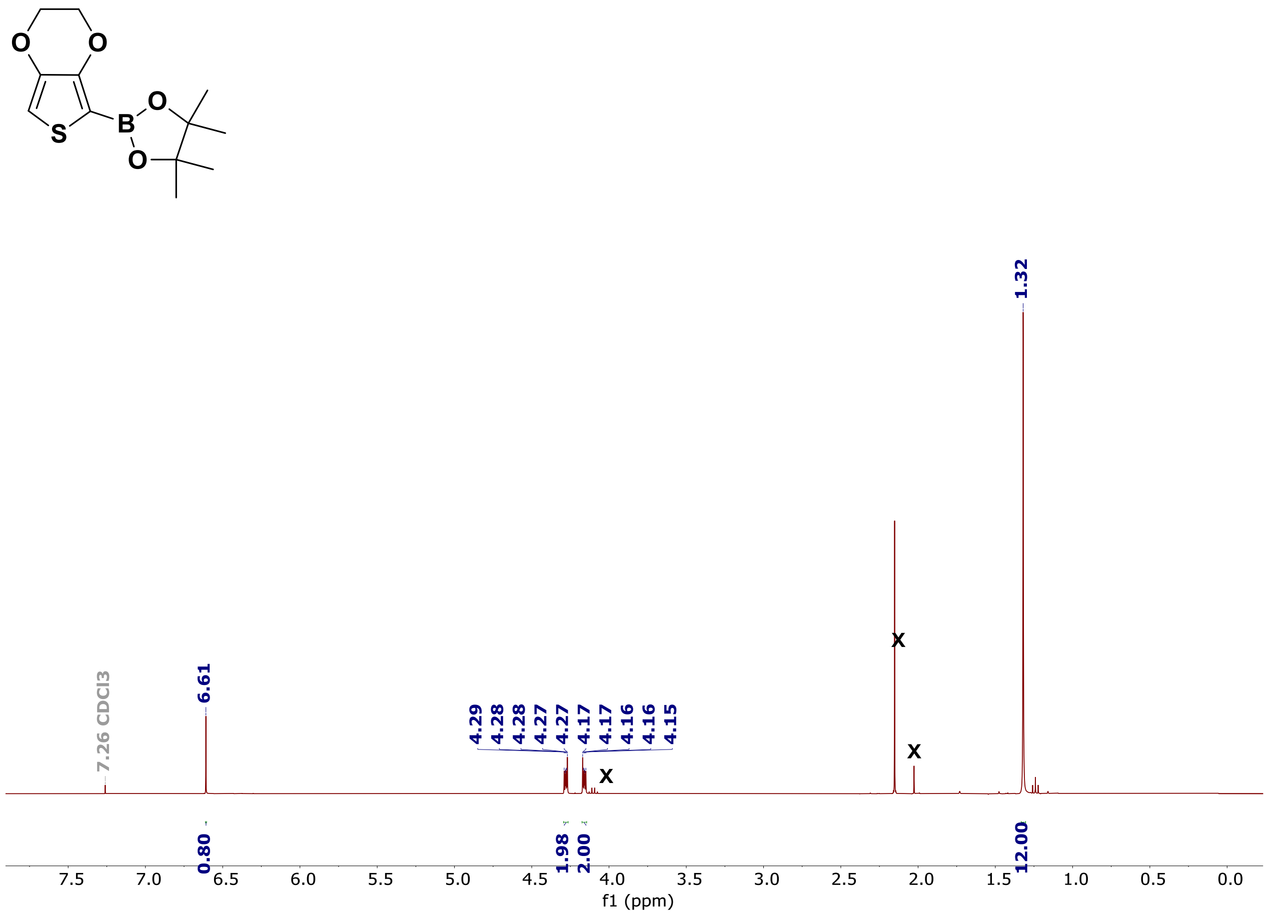

Figure S12: ^{1}HNMR of EDOT boronic acid pinacol ester (**4**)

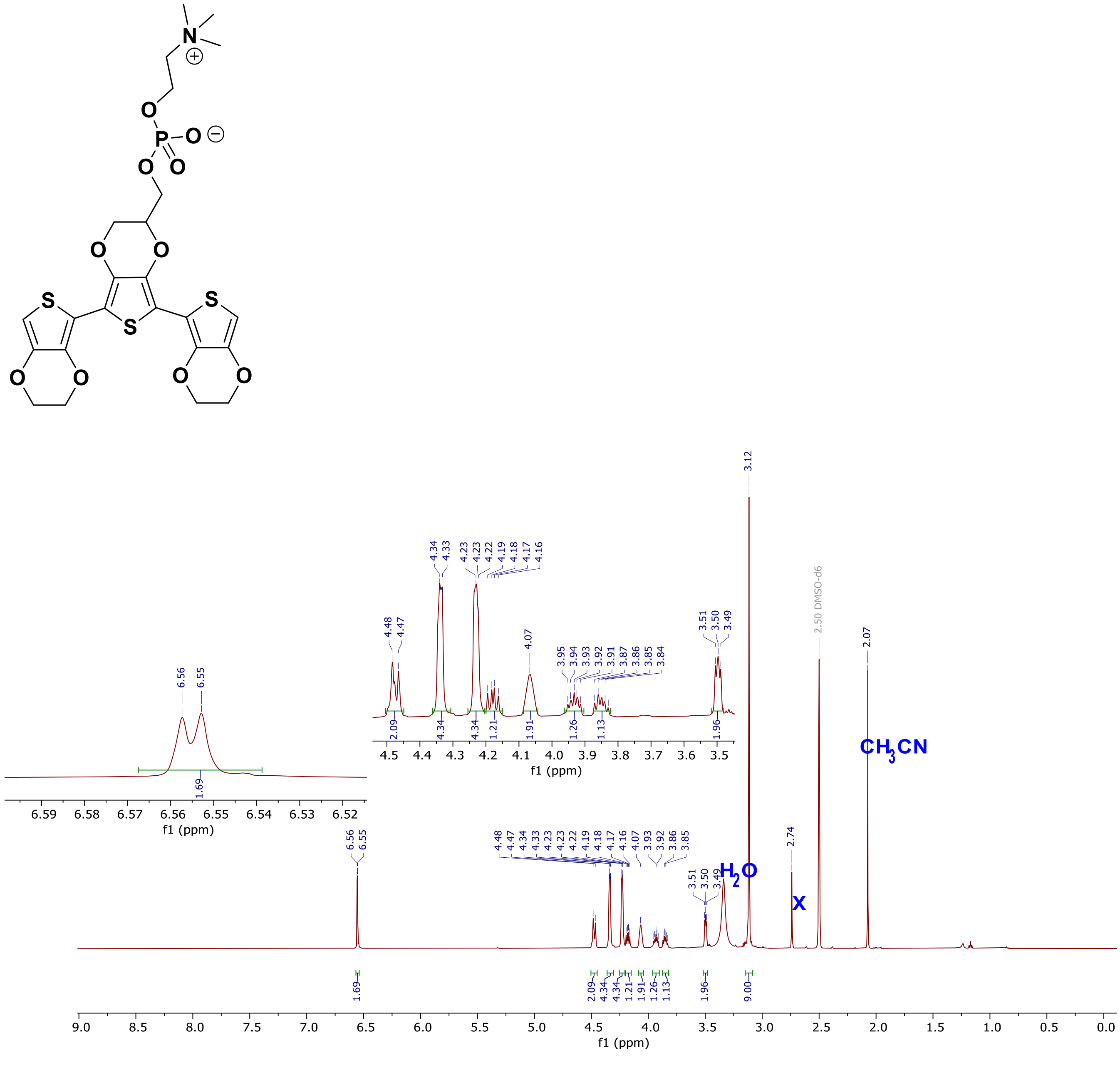

Figure S13: ^{1}HNMR of 3E-PC (**6**)

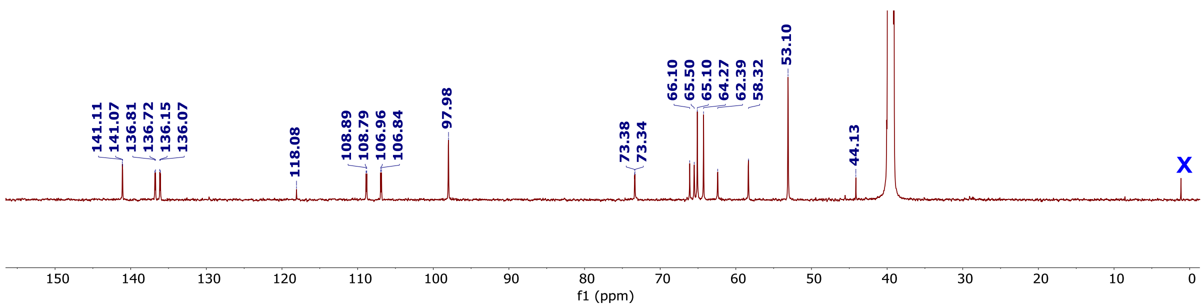

Figure S14: ^{13}CNMR of 3E-PC (**6**)

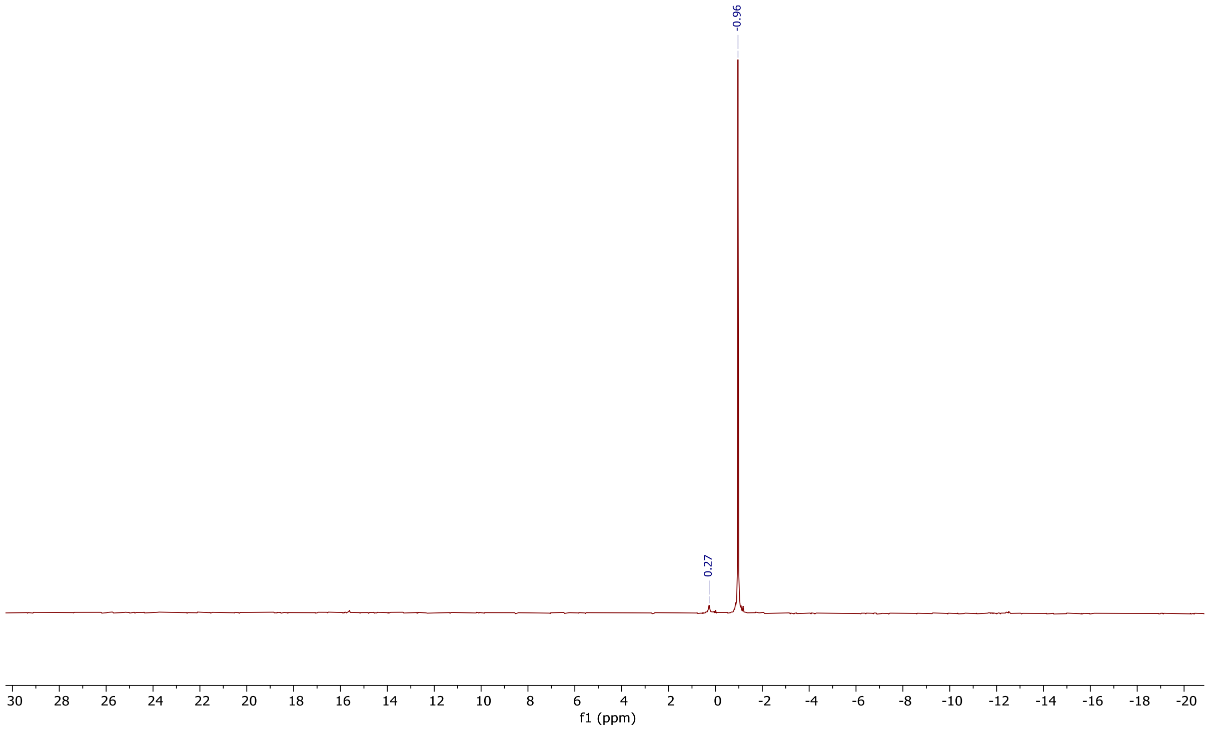

Figure S15:^{31}PNMR of 3E-PC (**6**)
